# Home visits for preterm/low birthweight infants in South Africa: Qualitative evidence synthesis

**DOI:** 10.4102/phcfm.v16i1.4701

**Published:** 2024-11-20

**Authors:** Sara Cooper, Idriss I. Kallon, Denny Mabetha, Amanda S. Brand, Tamara Kredo, Shakti Pillay, Gugu Kali, Willem Odendaal

**Affiliations:** 1 Cochrane South Africa, South African Medical Research Council, Cape Town, South Africa; 2 School of Public Health and Family Medicine, University of Cape Town, Cape Town, South Africa; 3 Department of Global Health, Stellenbosch University, Cape Town, South Africa; 4 Centre for Evidence-based Health Care, Division of Epidemiology and Biostatistics, Faculty of Medicine and Health Sciences, Stellenbosch University, Cape Town, South Africa; 5 MRC/Wits Rural Public Health and Health Transitions Research Unit (Agincourt), Faculty of Health Sciences, University of the Witwatersrand, Johannesburg, South Africa; 6 Health Systems Research Unit, South African Medical Research Council, Cape Town, South Africa; 7 Division of Clinical Pharmacology, Faculty of Medicine and Health Sciences, Stellenbosch University, Cape Town, South Africa; 8 School of Family Medicine and Public Health, Faculty of Health Sciences, University of Cape Town, Cape Town, South Africa; 9 Division of Neonatology, Groote Schuur Hospital, Department of Paediatrics and Child Health, Faculty of Health Sciences, University of Cape Town, Cape Town, South Africa; 10 Division of Neonatology, Department of Paediatrics and Child Health, Faculty of Health Sciences, Stellenbosch University, Cape Town, South Africa; 11 HIV and Other Infectious Diseases Research Unit, South African Medical Research Council, Cape Town, South Africa; 12 Department of Psychiatry, Faculty of Medicine and Health Sciences, Stellenbosch University, Cape Town, South Africa

**Keywords:** preterm and low birthweight (LBW) infants, maternal and child health, home visits, lay health worker, South Africa, acceptability, feasibility and equity, qualitative evidence synthesis

## Abstract

**Background:**

Prematurity and low birth weight (LBW) are the main causes of neonatal mortality in South Africa (SA). Home visits by lay health workers (LHWs) may be effective in addressing this.

**Aim:**

To inform a national guideline on LHW home visits as part of the Global Evidence, Local Adaptation (GELA) project, we conducted a rapid qualitative evidence synthesis exploring the acceptability, feasibility and equitability of this intervention for preterm and LBW babies.

**Setting:**

We included studies conducted in SA.

**Methods:**

We searched PubMed and Embase until 15 September 2023 and identified eligible studies independently and in duplicate. We synthesised evidence using thematic analysis, assessed study quality using an adaptation of the Critical Appraisal Skills Programme tool and assessed confidence in the review findings using GRADE-CERQual.

**Results:**

The 16 eligible studies included diverse settings and populations in SA. Factors facilitating mothers’ acceptance included the knowledge and skills gained, the psychosocial support offered and improved healthcare access and relationships with facility staff. Distrust in LHWs and stigma associated with home visits were barriers to acceptance. Lay health workers’ acceptance was facilitated by them feeling empowered. The emotional burden of home visits for LHWs, coupled with insufficient training and support, undermined the feasibility of home visits.

**Conclusion:**

A complex range of interacting contextual factors may impact on the implementation of home visit programmes for preterm and LBW infants in SA.

**Contribution:**

This country profile provides insights into how home visits for preterm and LBW infants in SA might be contextually tailored to increase local relevance and in turn effectiveness, with potential relevance for other African countries.

## Introduction

Over the last decade, South Africa (SA) has made significant improvements in maternal, neonatal and child health (MNCH) outcomes, primarily because of investments in human immunodeficiency virus (HIV) health services and prevention of mother-to-child transmission (PMTCT) programmes.^1^ It is estimated that the maternal mortality ratio (MMR) decreased from 173/100 000 in 2000 to 127/100 000 in 2020,^2^ under-five mortality rate reduced from 71/1000 in 2000 to 35/1000 in 2022^3^ and the neonatal mortality rate (NMR) dropped from 28/1000 live births in 2000 to 18/1000 live births in 2019.^4^ However, the country still struggles to reduce health inequalities and reach global MNCH targets.^5^ Equitable access to affordable high-quality MNCH services remains elusive to many communities in SA.^6^ Moreover, while the country has made progress towards reducing NMR, prematurity and low birthweight (LBW) rates have not improved significantly. United Nations International Children’s Emergency Fund-World Health Organization (UNICEF-WHO) estimates of LBW prevalence in SA were 17.2%, 16.6% and 16.6% in 2000, 2012 and 2020, respectively.^7^ Preterm birth complications, including LBW, is the largest cause of neonatal deaths in the country.^1,8^ The coronavirus disease 2019 (COVID-19) pandemic exacerbated the challenges, and early indications of its impact show dire effects on maternal and child mortality rates and the uptake of maternal services.^9^ A comparison of COVID-19 versus pre-COVID-19 periods showed a 40% increase in maternal deaths, with a 3% and 10% increase in neonatal mortality and stillbirths, respectively.^10^ Considering current resource constraints, it is unlikely that SA’s public health system will meet the demands for specialised care for preterm infants.^1,8^

There is evidence that community-based models of care, including home visits, may be well-positioned to positively impact MNCH outcomes.^11,12^ These interventions have been shown to be effective in reducing under-five mortality,^13,14^ rates of maternal depression,^15^ and improving access to healthcare,^16^ child growth and development outcomes.^17^

Since the 1990s, SA has seen a growth in community-based healthcare programmes, which has included various programmes to reduce maternal and child mortality and improve access to healthcare.^18,19^ In 2011, the National Department of Health (NDoH) launched the ‘Re-engineering of Primary Health Care’ policy, which included lay health workers (LHWs) to promote health and healthcare among pregnant women and mothers in their homes.^18^ The goal of this initiative, together with other similar interventions,^1,15^ is to bring appropriate care closer to mothers and babies to help close the service delivery gap in under-resourced communities. Despite good evidence that these types of programmes can positively influence a range of health outcomes, there are a myriad of challenges that impact their acceptability and feasibility, and in turn their effectiveness and scalability.^15,20^ A better understanding of these issues could provide important insights into how they can be better addressed in the design, implementation and scale-up of home visit interventions for families with preterm and LBW infants in the country.

Qualitative research is well-placed for exploring these complex issues, and the contexts in which they arise.^21,22^ Qualitative evidence synthesis (QES) – or systematic reviews of qualitative evidence – brings together the evidence from primary qualitative research in a systematic way.^23^ The findings from a QES can enable richer interpretations and more powerful explanations of phenomena, circumstances or experiences, than can be achieved by a single primary qualitative study.^24^ Qualitative evidence synthesis is increasingly being used within guideline development and policy formulation to incorporate evidence beyond the effects of interventions, to wider questions about local norms and preferences, equity and human rights issues, acceptability and feasibility of interventions, implementation processes and the impact of socio-political and cultural contexts.^21,22,25^

The Global Evidence, Local Adaptation (GELA) project aims to maximise the impact of research on poverty-related diseases through enhancing decision makers’ capacity to use global research to develop locally relevant clinical practice guidelines (CPGs) in the field of newborn and child health in SA, Malawi and Nigeria (https://africa.cochrane.org/projects/GELA). To help a national guideline development group (GDG) formulate a guideline recommendation around home visit programmes for families with preterm and LBW infants in SA, GELA sought to identify or produce contextually relevant qualitative evidence on the topic, along with quantitative evidence about intervention costs and effectiveness. Specifically, qualitative evidence was sought to inform judgements about the feasibility, acceptability and equity domains of the GRADE Evidence-to-Decision (EtD) framework, which is used to help decision-makers use evidence to make decisions in a structured and transparent way.^26^

Through our searches, we identified two relevant QESs with a global scope.^20,27^ The first^27^ provides important evidence on the values and preferences of families about the healthcare of preterm or LBW infants, but does not contain any evidence specifically on home visits as an intervention. The second relevant QES^20^ contains important insights into the barriers and facilitators to home visits to improve access to maternal and child health. However, we considered it to be out-dated (the searches were conducted in 2011) and lacking in qualitative evidence pertaining specifically to families of preterm and LBW infants. In discussion with the SA national GDG, we deemed it necessary to supplement these global QESs with more recent and more local qualitative evidence relevant specifically to home visits for preterm and LBW infants specifically for the SA implementation setting.

The aim of this rapid QES was therefore to: (1) synthesise evidence from qualitative studies investigating SA stakeholders’ views and experiences of home visit programmes for families with preterm and LBW infants in SA; (2) identify the factors influencing the acceptability, feasibility and equity implications of these programmes.

## Methods

### Search methods

We searched PubMed, Medline and Embase databases for eligible studies from inception up until 15 September 2023 (see Online Appendix 1 for the search strategies). We contacted experts, searched citation lists of included studies and key references and cross-checked studies in the linked effectiveness and economic reviews that were simultaneously conducted to inform the GELA national guideline recommendation.

### Inclusion criteria

We included studies that utilised qualitative methods for data collection and analysis; focussed on SA home visit programmes to improve health outcomes for preterm and LBW infants; and explored the views and experiences of any stakeholder involved in, or affected by, their design, receipt, delivery or implementation (e.g. any type or cadre of healthcare worker, patients and their families, peers, policy makers and programme managers).

For this review, we used the following definitions:

A home visit is an intervention where a trained health professional, LHW or volunteer visits the parents and/or caregivers of preterm and/or LBW infants in their home soon after discharge from hospital, to provide psychosocial support, health assessment, promotion and education, and referral services for problems identified. The frequency, duration and content of visits may differ by context.

A preterm infant is an infant born alive before 37 completed weeks of gestation,^28^ with further sub-divisions into moderate to late preterm (32–36 weeks), very preterm (28–31 weeks) and extremely preterm (less than 28 weeks).

Low birthweight refers to weight at birth of less than 2500 g.^29^ This could be further categorised into very LBW (< 1500 g) and extremely LBW (< 1000 g).

We were unable to identify any qualitative studies of home visits to improve health outcomes specifically for preterm and LBW infants in SA. We therefore broadened the scope of the review to include qualitative studies of home visit interventions or programmes to improve MNCH outcomes more broadly. In line with a global review on this topic,^20^ we used the following definitions in this regard:

Child healthcare is aimed at improving the health of children less than 5 years of age.

Maternal healthcare aims to improve reproductive health, ensuring safe motherhood, or is directed at women in their role as carers for children aged less than 5 years.

### Study selection

**Figure 1:**
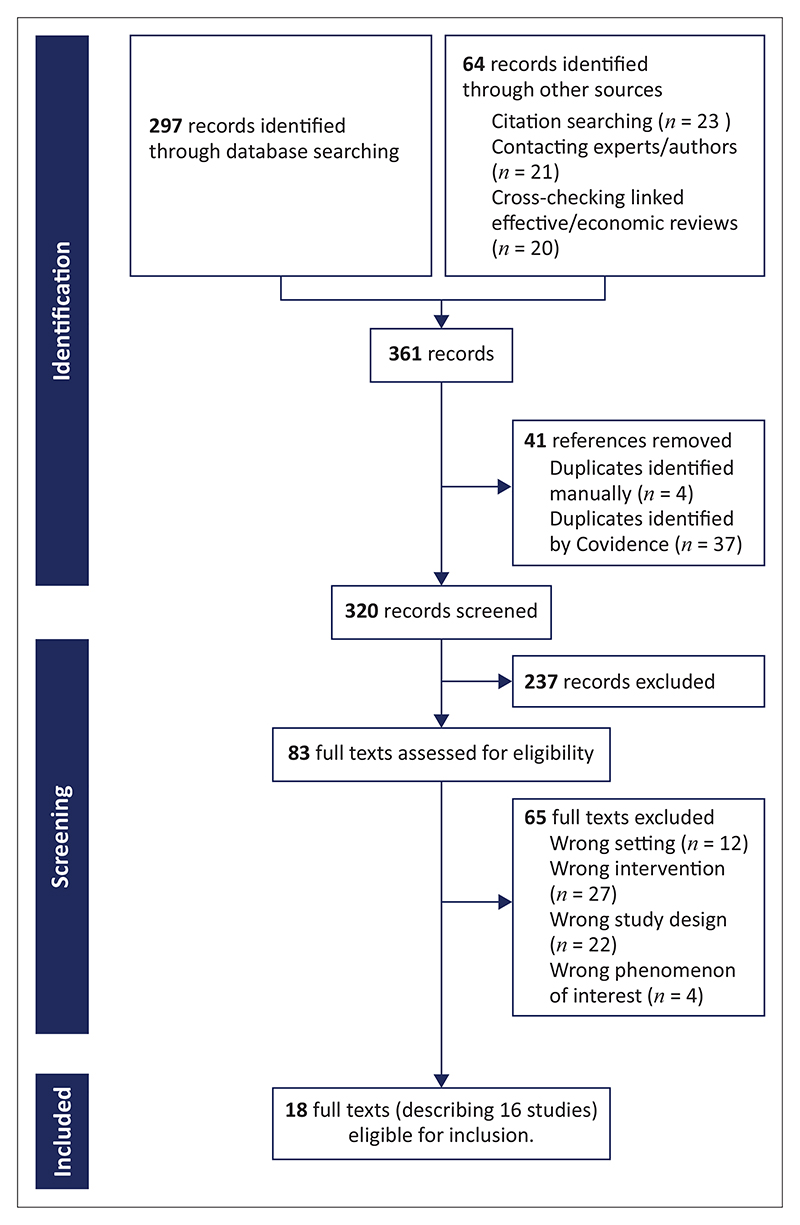
Study flow diagram. *Source*: Page MJ, McKenzie JE, Bossuyt PM, et al. The PRISMA 2020 statement: An updated guideline for reporting systematic reviews. BMJ. 2021. https://doi.org/10.1136/bmj.n71

Two review authors independently assessed the titles and abstracts of the identified records to evaluate eligibility. We retrieved the full text of all potentially relevant abstracts and assessed these papers independently and in duplicate. We resolved disagreements by discussion or, when required, by involving a third review author. Where the same study, using the same sample and methods, was presented in different reports, we collated these reports so that each study (rather than each report) was the unit of interest in our review. Figure 1 comprises a PRISMA (Preferred Reporting Items for Systematic Reviews and Meta-Analyses) flow diagram showing our search results and the process of screening and selecting studies for inclusion.

### Data extraction and analysis

We first extracted characteristics of the studies (citation, publication date, setting, duration, participants’ details, etc.) and thereafter its results. We used a thematic synthesis method as our analytical approach.^30^ Key concepts and themes (reported anywhere within the primary qualitative studies) were extracted for each study in Nvivo, together with supporting participant quotes. We also developed a brief, structured summary for each study, capturing the main conclusions. The extracted data, together with the structured study summaries, were then compared and contrasted across studies to identify commonalities and potential differences. To support this process and further organise the similarities and differences across studies, we drew on the constructs of the EtD framework for qualitative evidence – specifically seeking content on acceptability, feasibility and equity.^21,31^ Once the findings had been organised into themes, we re-read the included studies to check that all key study findings were captured by the review findings. One review author led the data extraction and analysis process, which was cross-checked by a second review author. The emerging findings were also discussed and workshopped with the other review authors.

### Study appraisal and confidence in the review findings

We assessed the methodological limitations for each study using criteria employed in previous Cochrane reviews.^32,33,34^ One review author conducted the assessment, which was cross-checked by a second review author. The criteria used were originally based on the Critical Appraisal Skills Programme (CASP) tool^35^ but they have since gone through several iterations. The adapted tool includes the following eight questions to assess methodological limitations:

1. Were the settings and context described adequately?
2. Was the sampling strategy described, and was this appropriate?
3. Was the data collection strategy described and was this appropriate?
4. Was the data analysis described, and was this appropriate?
5. Were the claims made/findings supported by sufficient evidence?
6. Was there evidence of reflexivity?
7. Did the study demonstrate sensitivity to ethical concerns?
8. Any other concerns?

We assessed how much confidence decision-makers and other users can place in individual review findings using the GRADE-CERQual (Confidence in the Evidence from Reviews of Qualitative research) approach.^36^ GRADE-CERQual evaluates confidence in a review finding based on four key components: (1) the adequacy of data supporting the review finding; (2) the relevance of the individual studies contributing to the review finding; (3) the methodological limitations of the individual qualitative studies contributing to the review finding; and (4) the coherence of the review finding. The assessment of each of these four components is then used to make a judgement about the overall confidence in the evidence supporting the review findings, which can be judged as high, moderate, low or very low. Two review authors applied GRADE-CERQual together, and the final assessment was based on discussion and consensus among all the review authors. All findings started as high confidence and were then graded down if there were important concerns regarding any of the GRADE-CERQual components.

Our GRADE-CERQual assessments and associated confidence in the review findings incorporated the fact that the body of evidence contributing to the review findings pertains to home visits and peer support in relation to MNCH more broadly, and not in relation to preterm and LBW infants’ health more specifically (i.e. ‘indirect’ evidence) (see Evidence Profiles – Online Appendix 2). In particular, and in line with the GRADE-CERQual approach, when making our assessments on relevance, we evaluated whether there are likely to be significant differences between preterm and LBW infants and infants that are not preterm and LBW that would reduce our confidence in relation to each review finding. To facilitate these judgements, we drew on the QES on what matters to families about the healthcare of preterm or LBW infants,^27^ and also consulted with experts on the topic of preterm or LBW infants in South Africa.

### Results of the search

No studies were identified that focussed on preterm and LBW infants specifically. In all, 16 studies – from 18 full texts – met our broadened inclusion criteria of MNCH (Figure 1).^37,38,39,40,41,42,43,44,45,46,47,48,49,50,51,52,53,54^ All included studies were published between 2010 and 2023. Details of the included studies are shown in Table 1.

### Review findings

We have organised the findings into four themes, which separate recipients’ and LHWs’ acceptance of home visits and the factors that influence this: (1) Facilitators of recipients’ acceptance of home visits; (2) Barriers to recipients’ and the broader community’s acceptance of home visits; (3) Facilitators of LHWs’ acceptance of home visits; (4) Barriers to LHWs’ acceptance of home visits and the feasibility of home visits. None of the studies identified or described potential impacts of home visits on equity issues explicitly, and thus we do not report any findings in this regard. We refer throughout to those delivering the intervention as LHWs.

Table 2, the summary of the qualitative findings (SoQF), provides a summary of each review finding, references to the studies contributing data to each finding and quotations as supporting data. It also details our assessment of confidence in the evidence, as well as an explanation of this assessment, based on the GRADE-CERQual approach.^36^ Detailed descriptions of our confidence assessment are included in the evidence profiles in Online Appendix 2. Our assessment of the methodological strengths and limitations of included studies is included in Online Appendix 3.

## Theme 1: Facilitators of recipients’ acceptance of home visits

The studies found the following aspects about home visits that mothers value and which may facilitate their acceptance of them: (1) Acquiring knowledge and skills; (2) Time to learn and express needs; (3) Psychosocial support; (4) Reduced clinic visits; (5) Better access to, and relationships with, clinics and clinic staff; (6) LHWs coming from same community; (7) Incorporating innovative digital technologies; and (8) Mothers reported positive impacts of these different elements.

### Finding 1: Acquiring knowledge and skills (High confidence)

Many mothers expressed appreciation of the educational element of home visits, whereby through information provision, hands-on activities and problem-solving they learnt new information about their own and their children’s health. Some mothers described how the practical advice and information they received from LHWs was relevant to their lives and also conveyed in a way that they could easily understand.

### Finding 2: Time to learn and express needs (High confidence)

Many mothers emphasised that home visits provided them the opportunity to have more time with a healthcare provider, allowing them to express their needs and discuss different topics without time constraints. Some contrasted this with their experiences of healthcare facilities, explaining how staff in these settings are usually extremely busy and lack adequate time for consultation. Many mothers also described feeling anxious when attending clinics and finding it difficult to ask questions, often leaving the clinic feeling that they had not received the information or treatment they required. In contrast, many mothers appreciated what they described as LHWs’ patience during home visits and their willingness to take the time to explain important topics while addressing their questions and concerns.

### Finding 3: Psychosocial support (High confidence)

Some mothers highlighted the importance of the supportive role played by home visits. Specifically, they spoke about the emotional support LHWs provided, allowing them to discuss their problems and listen to them with compassion and respect. Many spoke about how LHWs helped alleviate their sense of loneliness and alienation or offered them hope and encouragement when they felt hopeless. Mothers also highlighted the responsiveness of LHWs, often available to help after hours and when a problem arises. Some mothers appreciated the continuity of their interactions with LHWs, which they felt created a support structure that they could rely on.

### Finding 4: Reduced clinic visits (Very low confidence)

Some mothers described how home visits reduced the number of clinic visits needed, with the resultant time, convenience and cost savings they experienced. Many mothers indicated that clinic visits were expensive and time-consuming, often requiring them to travel very early in the morning and spend the whole day at the clinic. This was seen as a major challenge for accessing healthcare.

Some LHWs similarly described how the mothers they visited often highlighted the many challenges they face in accessing healthcare facilities, including practical issues of distance and transport, childcare constraints and competing household work pressures. They highlighted how mothers frequently indicated how they appreciated being visited at home because of the time, effort and (opportunity) costs this saved them.

### Finding 5: Better access to, and relationships with, clinics and clinic staff (moderate confidence)

Many mothers spoke about how home visits enhanced their access to, and relationships with clinics and clinic staff; for example, they described how access to clinics could be facilitated by LHWs providing them with a signed referral letter for clinic staff. Other mothers spoke about the communication channel LHWs provided, giving them helpful information on how to efficiently navigate clinic appointments. Some mothers felt that home visits empowered them as a result of the knowledge and skills provided, which in turn helped them to feel more confident and equipped to communicate with nurses and ask questions.

### Finding 6: Lay health workers coming from same community (Moderate confidence)

For some mothers, acceptance of home visits appeared to be facilitated by LHWs from the same community; many described how this made LHWs more familiar and approachable and/or able to understand their lived experiences and contexts better. This enhanced the trust between many mothers and LHWs. In addition, some mothers appreciated that LHWs from the same community were in close proximity and therefore easily accessible in an emergency. Some LHWs also indicated a preference for working with mothers within their own communities, suggesting that this facilitated trust and relationship-building and in turn acceptance among mothers. Many LHWs described this as essential for their work.

### Finding 7: Incorporating innovative digital technologies (Very low confidence)

Two studies explored the impact and experiences of incorporating digital devices containing mobile video content as health promotion and teaching tools during LHW home visits.^37,39^ Both studies found that digital tools were highly valued by mothers, which also enhanced their acceptance of home visits. Some LHWs felt that video messages assisted with capturing mothers’ attention and maintaining their interest. Many LHWs reported that mothers would at times spontaneously express interest in watching additional or new videos on arrival at their household. Many LHWs felt that the video messages underscored and legitimised the advice they provided. Because the videos echoed the early perinatal health messages that mentor mothers delivered prior to video viewing, LHWs felt that the mothers they counselled were more likely to trust them and value their expertise. They also thought that the digital technologies themselves enhanced their perceived authority in the community by signifying that they were employed by a well-funded, well-established programme, and by allowing them to demonstrate technological skills in front of the mothers they visited.

### Finding 8: Perceived positive psychological and behavioural impact (Moderate confidence)

Many mothers described several positive effects they perceived to have experienced because of the above-mentioned elements of home visits. For some, these visits reportedly facilitated behaviour change through the knowledge and skills gained, or through the sense of external accountability they felt from having a monthly or bimonthly visitor whom they respected. Others described gaining a sense of agency and confidence in health-related decision-making, even when they were unable or unwilling to fully adhere to the advice. Many mothers described feeling empowered by the home visits, which encouraged them to take better responsibility for their own health and that of their child’s. Some mothers described feeling a greater sense of well-being and hope because of the positive outcomes that they had observed in themselves or their children, and the fact that they had received something worthwhile.

## Theme 2: Barriers to recipients’ and the broader community’s acceptance of home visits

The studies revealed various barriers to acceptance of home visits among mothers and the broader community. Here two findings emerged as particularly prominent: (1) Distrust of LHWs for several reasons; and (2) Stigma associated with home visits.

### Finding 9: Distrust of lay health workers (High confidence)

Distrust of LHWs emerged as a major overarching factor contributing to mothers’ and the broader communities’ reservations of home visits. The reasons for this distrust included issues related to (1) Privacy and confidentiality; (2) LHW gender; and (3) Perceived competencies of LHWs.

### Finding 9.1. Privacy and confidentiality (Moderate confidence)

Some mothers distrusted LHWs because of concerns related to privacy and confidentiality. These mothers indicated that LHWs could not be trusted as they did not maintain client confidentiality, reportedly gossiping or disclosing private information to others in the community. This was perceived to be a particularly significant concern when LHWs came from the same community as mothers. It was also seen as a concern when LHWs used digital devices, as found by one of the studies exploring the impact and experiences of incorporating digital device tools during home visits.^39^ Many LHWs reported that some clients were concerned that the devices were being used as voice or video recorders, which was found to be an integral component of some mothers’ more general concerns regarding LHWs, in terms of maintaining their privacy and confidentiality.

The home, as a communal consultation space for receiving services, was reported to present further challenges to privacy and confidentiality. Some mothers and LHWs indicated how discussing confidential information at home was challenging if family members were present and could lead to unwanted disclosure of sensitive information. Both mothers and LHWs explained how visits sometimes caused contention or led to curiosity from family members, which undermined the trust relationship between the LHW and the mother.

### Finding 9.2: Gender of the LHW (Very low confidence)

Another potential factor contributing to mothers’ distrust of LHWs is related to the gender of the LHW. In respect of male LHWs, it was suggested that mothers may be concerned for their safety and may be doubtful about what they could teach them. It was also suggested that some male partners of women being visited expressed concerns about a man discussing topics that were perceived to be of a sensitive and personal nature. This was thus an additional factor potentially reducing the acceptability of male LHWs visiting households in the antenatal and postnatal periods.

### Finding 9.3: Perceived lack of competencies of lay health workers (Moderate confidence)

An additional factor contributing to maternal distrust is related to LHWs’ perceived lack of competencies as healthcare service providers. Some studies revealed various factors that could undermine recipients’ confidence in LHW competencies. The relationship between LHWs and clinic staff was one such factor, with studies showing how clinic staff were crucial in supporting confidence in LHWs; if clinic staff appeared to question LHWs’ competency or trustworthiness, this could undermine LHW credibility in the eyes of the community.

While some LHWs described trusting and good working relationships with clinic staff, others reported clinic staff treating them with contempt and disrespect, and conveying a lack of confidence in their ability to provide appropriate services. Lay health workers provided examples in this regard, such as clinical staff not accepting their referrals, not taking them seriously, or not drawing on them to help in busy times. Many LHWs felt that this treatment affected their perceived competency by the community and undermined community trust in their ability to provide care.

Other factors identified as potentially undermining confidence in the competency of LHWs included individuals in wealthier households with potentially higher educational levels than LHWs undermining their role; perceptions of LHWs as voluntary and temporary workers occupying a lower status than nurses; and poor LHW performance and access to essential medical equipment.

### Finding 10: Stigma associated with home visits (Moderate confidence)

In addition to distrust, stigma associated with home visits was an additional factor undermining its acceptance among mothers and the broader community. Many LHWs indicated that certain people in the community had little knowledge of what their work entailed and believed that they only visited people living with HIV, which led to stigma regarding home visits. Other LHWs suggested that there was stigma surrounding perceptions that they only visit mothers who are emotionally struggling, and that some mothers feared they would be judged if they received home visits.

## Theme 3: Facilitators of lay health workers’ acceptance of home visits

The studies reported two issues that improved LHWs’ acceptance of home visits: (1) it offered them a sense of empowerment; and (2) they enjoyed using digital technologies during the visit.

### Finding 11: Empowering, validating, employment and convenience (Moderate confidence)

A number of studies indicated in general terms that LHWs tended to be supportive of home visits and their associated role in them. Some reasons were provided, although details were relatively lacking in this regard. These included the sense of empowerment, dignity, purpose and strength LHWs gained from being respected as healthcare workers in their community. Some LHWs felt valued as individuals who were seen to be able to make a difference for their community, while others reported seeing the impact their visits make. This provided them with a sense of value and worth. Many described feeling pride in earning a salary. A few spoke about the added benefit and convenience around being able to work within their own community, where many residents seeking formal employment are forced to migrate and live away from their families.

### Finding 12: Incorporating innovative digital technologies (Very low confidence)

The two studies that explored the impact and experiences of incorporating digital devices during home visits (Finding 7) found the devices were highly valued by LHWs.^37,39^ This enhanced their acceptance of their home visiting role more generally. Many LHWs felt that the digital devices lightened their workload as they did not need to perform all health counselling verbally and could therefore focus on other important tasks. For example, they highlighted how the use of videos allowed them to perform other administrative- and health-related tasks, such as note-taking or completing referrals while mothers engaged with the videos. Many LHWs also reported that they felt these devices helped to convey a sense of importance about their work, which boosted their confidence, morale and sense of self-worth.

## Theme 4: Barriers to lay health workers’ acceptance of home visits and the feasibility of home visits

The studies revealed various challenges LHWs face when conducting home visits which may reduce both their acceptance and feasibility of home visits. These challenges are grouped into four findings: (1) Boundaries and burdens; (2) Training, supervision and support; (3) Practical and logistical challenges; and (4) Human resource-related issues.

### Finding 13: Boundaries and emotional burdens (High confidence)

Many LHWs spoke about the difficulties they experience in maintaining boundaries and navigating between professional and personal obligations. This was particularly the case when LHWs came from the same community as the mothers they visit. In such cases, LHWs indicated that they have less ability to draw boundaries because they are always available for their clients. Some also felt obligated to attend to mothers’ needs after hours. Lay health workers described how the realities of poverty and community violence they confront daily made it at times difficult to maintain the boundaries of the programme, and what they are meant to provide.

Some LHWs described finding it difficult to separate themselves from their clients’ problems, particularly when they shared similar burdens (e.g. HIV, poverty and crime), or when they felt emotionally connected to clients. They described this as distressing and painful, and taking a toll on their emotional well-being.

Lay health workers described various other burdens they face. Many noted that being close to mothers invited the possibility that gossip about someone’s health could be pinned back on them, even if they were not to blame. Some spoke about the jealousy they experience from community members because of being employed in a context of low employment rates. A few described how their LHW role had disrupted the dynamics in their homes, because of shifts in cultural expectations with, for example, the financial independence they had gained threatening their husbands.

### Finding 14: Training, supervision and support (High confidence)

Gaps in knowledge and expertise were identified as a major challenge across LHW home visit programmes albeit to varying degrees. Many LHWs expressed concern that they did not have the necessary information and skills to provide the recommended care. They felt that this deficit undermined their performance during home visits and impacted on their perceived credibility by the mothers under their care.

This finding was similarly identified in studies evaluating LHW practices where it was noted that many LHWs displayed important gaps in knowledge and expertise. These studies found that LHWs did not always provide all essential content or perform the practices considered critical for their routine activities. One study evaluated the fidelity of core intervention skills taught in training LHWs.^46^ The study found that, while the core knowledge and skills that were taught were widely observed among LHWs, the more complex interpersonal skills (e.g. soliciting questions or reflecting clients’ feelings and concerns) were not readily observed across all LHWs or during all visits.

This deficit in knowledge and skills was attributed to inadequate training that LHWs receive, together with poor supervision and support. While some LHWs appeared to be positive about their initial training, many felt that they had received insufficient refresher training. Moreover, LHWs reported operating largely in isolation, with limited access to the support and supervision needed to carry out their work effectively. In particular, some expressed dissatisfaction with the limited in-field supervision they received, with supervision reportedly often being conducted at clinics or on the phone.

Three studies explicitly emphasised the importance of training and supervision for LHWs.^40,44,51^ In one study assessing the impact of an intervention package, it was found that ongoing support, and increased supervision and training, enhanced LHW knowledge and confidence, increased motivation and a sense of accountability among LHWs.^44^ In another study, it was found that LHWs were initially unhappy with the intervention as they felt it would add more work.^51^ However, this perception changed after LHWs received increased supervision and mentorship. Lay health workers in this study described the many positive effects of this supervision and mentorship, including increased knowledge, motivation and confidence. Many also detailed how mentors accompanying them during the home visits improved their communication with mothers. In the third study, it was found that strong supervision and support structures for LHWs were essential for them to carry out their work.^40^ It was revealed that such support helps LHWs to manage the stress and emotional toll of home visits. Specifically, this study found that, along with technical and informational supervision, other types of supportive supervision for LHWs are required. These include, for example, mentoring and motivating LHWs; managing the administrative, emotional, and safety demands of home visits; and helping them to set boundaries and engage in self-care practices.

### Finding 15: Practical and logistical challenges (High confidence)

Some LHWs provided detailed descriptions of the range of practical and logistical challenges they face during home visits, which reportedly make it difficult for them to perform their work successfully. Distance was a common challenge, with many LHWs describing how they often covered vast distances between households, frequently with limited or no access to transportation.

Another challenge was the mobility of mothers; for example, some mothers living in informal settlements relocated frequently. This made following-up with these mothers difficult, especially if the mother had moved to another LHW’s area. Mothers also frequently visited the father of the child or sought work in other areas and were therefore not available for home visits.

Another major challenge commonly shared by LHWs was personal safety. Many reported feeling unsafe when visiting mothers residing in areas prone to violence, crime and drug abuse. Some LHWs described being bitten by mothers’ dogs and occasionally having to cancel a home visit because of dogs impeding access to the home.

Some LHWs often had limited access to essential tools and equipment to conduct their work. They felt that this strongly hindered their ability to perform tasks and undermined programme credibility, and in turn community acceptance. In the study that assessed the impact of an intervention package to increase LHW support and training (Finding 14), LHWs were provided with extra equipment (e.g. scales, backpacks and phones), and access to transport.^44^ This was found to enable LHWs to deliver improved services, which in turn had a positive effect on perceived programme credibility and community acceptability.

### Finding 16: Human resource-related issues (Moderate confidence)

Some studies showed that human resource-related issues, including poor salaries, undermine the feasibility and LHW acceptance of home visits; many LHWs were reportedly unsatisfied with their remuneration. Non-permanent contracts for LHWs and the associated vulnerability were additional issues identified. These factors contributed to high LHW attrition rates.

## Discussion

In this review, we aimed to synthesise qualitative evidence on stakeholders’ views and experiences of home visit interventions for families of preterm and LBW infants in SA. A noteworthy result was that no studies were identified that focussed specifically on preterm and LBW infants. We therefore broadened the inclusion criteria of this review to include home visits for MNCH more broadly. The results of this ‘indirect evidence’ therefore need to be viewed with some degree of caution. More research specifically on preterm and LBW infants in SA is needed so that more definitive conclusions can be made regarding the acceptability and feasibility of home visits for this particular population.

None of the studies identified or described potential impacts of home visits on equity issues explicitly, and thus we did not report any findings in this regard. When developing the equity domains of the GRADE EtD framework to inform the SA national guideline recommendation, we used the findings from this review to infer potential impacts of home visits on equity issues. These hypotheses were, however, not included in the findings of this review as they did not emerge directly from the results reported in the primary studies. Again, more research on home visits for preterm and LBW infants in SA is required and particularly the equity implications of this intervention.

The findings of this review revealed a mix of acceptable intervention aspects and reservations among recipients and providers. Many mothers appreciated home visits for various reasons, including the knowledge, skills and psychosocial support it provided, the time it afforded them to learn and express their needs. In addition, home visit had various positive effects on mothers’ relationships with healthcare facilities which included reducing the number of visits needed, enhancing access and improving interactions with clinic staff. However, the findings also revealed various factors that may hinder mothers’ acceptance of home visits, including concerns related to privacy and confidentiality, LHW gender, perceived competencies of LHWs, associated distrust of LHWs and potential stigma associated with home visits.

A similar mixed picture emerged in relation to LHWs’ acceptability of home visits. Data on LHWs’ acceptance of home visit programmes and its facilitators were relatively sparse across the studies. It is unclear whether this reflects limited LHW acceptance of home visits or a tendency of studies to focus more on barriers and challenges. That said, the findings did suggest that many LHWs were supportive of home visits, potentially because of the empowerment, validation and the convenient employment it affords. However, the studies revealed a plethora of challenges LHWs face when conducting home visits which may reduce their acceptance and the feasibility of home visits. These included the emotional toll many LHWs face maintaining boundaries with clients and navigating between professional and personal obligations, the inadequate training, supervision and support they receive, and the multiple practical and logistical challenges they experience which make it difficult to perform necessary tasks and maintain personal safety.

The challenges to the acceptability and feasibility of LHW home visit programmes identified in this review are not new nor unique to SA. A global QES conducted over a decade ago on LHW programmes to improve access to maternal and child healthcare revealed very similar barriers.^20^ The review similarly found that, while recipients were generally positive about the programmes, many had concerns about confidentiality when receiving home visits, and questioned the competencies of LHWs. As in our review, this global QES also found that LHWs sometimes find it difficult to manage emotional relationships and boundaries with recipients and face a range of health system constraints related to inadequacies of equipment, support, training and remuneration. And similar to our review, this QES found that these challenges negatively impact on LHWs motivation, their credibility and programme success. These findings were similarly revealed in a recent QES about LHWs’ experiences and perceptions of supervision in programmes targeting maternal and child health in low-and-middle-income countries (LMICs).^55^ The review found that regular, good-quality training and in-the-field supervision are essential for programme effectiveness, and yet are missing in practice in most maternal and child health LHW programmes in LMICs.

The systemic challenges identified in our QES also appear to be similar to the issues facing LHW programmes in SA more broadly and not uniquely with regards to maternal and child health. Lay health workers and community-oriented care, including LHW home visit programmes, have over the past decade emerged as core elements of SA’s healthcare system, as captured in the 2004 CHW National Policy Framework, and more recently in national strategies such as the re-engineering of primary health care,^56^ the National Health Insurance (NIH)^57^ and ward-based outreach teams (WBOTs).^58,59,60^ However, research on these initiatives has revealed that LHW programmes in South Africa, including home visits, are often fragmented, inadequately planned, poorly supervised, under-resourced and not prioritised politically.^59,61,62^ These issues are often driven by well-intentioned vertical programmes that are inadequately integrated with facility-based primary health care service delivery^63^ and do not take the complexity of community-orientated primary care (COPC) into account.^64^ Adding home visits for LBW and preterm infants and their families as another vertical programme – rather than as part of an integrated primary health care system – risks fuelling similar challenges. Many questions therefore remain regarding the feasibility to scale and sustain LHWs programmes more broadly, and not just in relation to maternal and child health, in South Africa. We attempt to address these questions in Table 3 and the implications for policy and practice in the next section.

### Implications for policy and practice

Table 3 includes a set of questions and prompts that may help policy makers and other decision-makers when planning, implementing or managing LHW home visit programmes for families with preterm and LBW infants in SA. These questions were developed by drawing on and integrating the findings from this review with (1) the implementation considerations that were developed out of the global review of qualitative research,^20^ now published as a policy brief^65^; (2) the principles identified by a scoping review of COPC in Africa, including different models and their effectiveness and feasibility^64^ and (3) input and discussion among the SA national GDG.

### Limitations

Because of the time constraints for developing the SA national guideline, this was a rapid review with associated limitations. We only searched two databases; a more comprehensive search of additional databases, including those not specific to health, may have identified more studies. Only one review author primarily conducted the data analysis process, albeit with discussion and input from other review authors. We recognise that qualitative data analysis is inherently interpretive; more than one review author undertaking these processes independently may have produced different interpretations and enhanced the exploration of alternative explanations. The assessments of methodological strengths and limitations were also undertaken by one review author, albeit checked by a second review author. More than two review authors conducting the assessments independently may have enhanced the rigour of the process.

## Conclusion

The findings from this review suggest that a range of complex and interacting contextual factors may impact on the acceptability and feasibility of home visits for maternal and child health in South Africa. In an attempt to address some of these factors visits, we have provided a set of questions that may help policy and decision-makers when planning, implementing or managing such programmes for preterm and LBW infants specific to the South African context.

## Supplementary Material

Appendices

## Figures and Tables

**Table 1:** Characteristics of included studies (16 studies from 18 full texts).

Study#	Reference	Programme / intervention	Setting (incl. data collection date)	Aim of study (as reported in the papers)	Participants	Methods
**Home visits**						
1	^37^	**Philani Mobile Video Intervention for Exclusive breastfeeding (MOVIE) study.**13 short (2 min – 5 min) teaching videos administered by mentor mothers during their regular perinatal home visits.	Khayelitsha, Cape Town; Western Cape 2019–20	Establish the effectiveness of the Philani MOVIE intervention and characterise, using a nested, qualitative performance evaluation, the acceptability and desirability of the intervention, as well as the mechanisms of action.	Mentor mothers (*n* = 26; 15 from the video intervention group and 11 from the control group)	In-depth interviews
2	^38^	**Ububele Mother-Baby Home Visiting project.**Lay home visitors trained (54 h) in a psychoanalytic and attachment-informed infant mental health theory that promotes a relational model of infant development. They provide an intervention (home visits over 14 weeks) that supports high risk mother–infant relationships in the same locality.	Alexandra, Johannesburg; Gauteng data collection date unclear	Explore convergences and divergences between current research-based, relational IMH models and ‘community’ knowledge held by a group of South African lay home visitors.	4 lay home visitors who were trained to conduct home visits	Semi-structured interviews subsequent to lay home visitors training and after 9 months working
3	^39^	**Philani Health and Nutrition Project + tablets with teaching videos.**Tablets with teaching videos (about HIV, alcohol, nutrition and breastfeeding) developed to support the health promotion efforts of mentor mothers who form part of the Philani Health and Nutrition Project in Khayelitsha. Each LHW visits approximately four community members each day with a total caseload of 50–80 families.	Khayelitsha, Cape Town; Western Cape Nov15–May16	Explore the acceptability and feasibility of using tablets with teaching videos (about HIV, alcohol, nutrition and breastfeeding) to support the health promotion efforts of Mentor Mothers who form part of the Philani Health and Nutrition Project in Khayelitsha.	24 Mentor mothers	Focus group discussions (FGDs)
4	^40^	**Promise EBF study.**Infant feeding peer counsellors. Local women employed to provide community peer counselling on infant feeding to mothers; one antenatal support visit, followed by postnatal support visits in weeks 1, 4, 7 and 10.	Rietvlei and Umlazi (KwaZulu-Natal [KZN]), Paarl (Western Cape); conducted 2006	Explore the experience of three LHW supervisors who were responsible for supporting infant feeding peer counsellors.	3 supervisors, each had between 10 and 12 peer counsellors.	Semi-structured interviews
5	^41^	**KZN DoH LHW MCH** **programme.**LHWs employed by the South African DoH in KZN-received 2-week training to develop the skills to provide care and support to pregnant women, mothers, newborns and children in the community	Primary health care clinics in five rural districts in KZN; data collection date unclear	Explore the acceptability of LHWs conducting household visits to mothers and infants during pregnancy and after delivery, from the perspective of community members, professional nurses and LHWs themselves.	65 caregivers (mother, father or grandmother of a child aged under 5 years); 37 professional nurses; 41 LHWs	19 FGDs with caregivers, LHWs, Professional nurses
6	^42^	**Mentor Mothers Zithulele (MMZ).**Mentor Mothers (MMs) undertake door-to-door visits in the local community to provide mothers regular check-ups together with support on health, nutrition and childcare, based on the Philani Mentor Mother model.	Zithulele village, remote rural district in Eastern Cape; data collection date unclear	Explore participants’ experience of the impact of peer mentoring on the rural communities they serve.	HIV-positive women participating in MMZ (*n* = 14) and women receiving standard PMTCT care without any intervention (*n* = 11); Mentor mothers (*n* = 8)	Semi-structured interviews with HIV-positive women participating in MMZ and a focus group discussion with the Mentor mothers delivering the intervention
7	^43^	**Eastern Cape Supervision Study**which assessed whether an enhanced supervision package delivered to government employed LHWs in the rural Eastern Cape, South Africa improved maternal and child outcomes when compared to routine supervision as delivered within the primary health care system. Although LHWs serve their communities at large, ECSS focussed specifically on pregnant women, mothers, and young children. The enhanced supervision package comprised additional training, resources and both administrative and supportive supervision, based on the Philani Mentor Mother model.	O.R. Tambo District, rural Eastern Cape; June and July 2021	Explore the status of supervision in the government-implemented LHW programme and experiences of the enhanced supervision package delivered as part of the ECSS	Mid-level supervisors involved in eight rural clinics enrolled in both the intervention and control arms of the RCT titled ECSS. Participants from three levels of clinic and non-governmental organisations: nine government-employed LHW supervisors or clinic personnel, two RCT programme managers and two RCT LHW supervisors.	Semi-structured qualitative interviews (*N* = 14) analysed thematically
8	^44^	Same intervention as ^43^	O.R. Tambo District, rural Eastern Cape; June and July 2021	Explore experiences of the enhanced supervision package delivered as part of the ECSS 3 months’ post-last follow-up in the trial	Eight LHWs from each arm of the RCT, and two supervisors from the intervention arm (total *n* = 18).	Semi-structured qualitative interviews (*N* = 18) analysed thematically
9	^45,46^	**Enable Mentor Mothers Programme,** a ‘social franchise’ of the Philani Mentor Mother model, a home-based prevention programme implemented by the Philani Maternal, Child Health, and Nutrition Project. In Philani’s model, MMs or ‘positive peer deviants’ are trained to serve as paraprofessional community health workers for home visiting among pregnant women and their families. Specifically, MMs conduct house-to-house visits on foot, enrolling pregnant women as well as undernourished children as clients. Pregnant clients are followed up throughout pregnancy as well as afterwards for up to 6 years. The programme’s key focus areas include maternal and infant wellbeing, child nutrition, immunisation, HIV/AIDS prevention and treatment, and access to social and health services. MMs also identify and visit undernourished children and clients with chronic conditions requiring home-based care. Each LHW visits approximately four community members each day with a total caseload of 50–80 families.	Nyandeni, rural part of Eastern Cape Province; Conducted February-March 2018	Explore LHW fidelity (content and structure of delivery) of the home-based maternal and child health intervention.	Clients of the programme and LHWs	Audio recordings of LHW home visits (*n* = 84). Themes across transcripts analysed through the newly developed Home Visit Communication Skills Inventory (HCSI)
10	^47,48^	Same intervention as in^45^	Nyandeni, rural part of Eastern Cape Province; conducted February–March 2018	Explore clients’ views and experiences of the Enable Mentor Mother programme and their engagements with LHWs	Pregnant or recently delivered clients (*n* = 26) of the Enable Mentor Mother programme	Individual interviews (*n* = 26) analysed thematically
11	^49^	Same intervention as in^45^	Nyandeni, rural part of Eastern Cape Province; conducted February–March 2018	Explore LHWs or MMs views and experiences of the Enable Mentor Mother programme	Mentor Mothers (*n* = 10)	Individual interviews (*n* = 10) analysed thematically
12	^50^	Same intervention as^43^	O.R. Tambo District, in rural Eastern Cape province	Understand the LHWs’ experiences of becoming and working as LHWs in the government-implemented LHW programme prior to initiating the ECSS	LHWs enrolled in the intervention arm of the ECSS	Semi-structured qualitative interviews (*n* = 9) and focus groups (*n* = 2) analysed thematically
13	^51^	**Family MUAC Project,** anchored in the Side-by-Side campaign. Facilitating a 1-day training for all LHWs in each of the sites to refresh LHWs skills in nutrition counselling, MUAC measurement and LHW’s implementation of the family MUAC in the household. Identification and lists of all households served by participating LHWs where there were children aged between 6-months and 5 years + Planning of Nutrition Health Days to raise awareness of the importance of nutrition and growth monitoring and introduction of the MUAC measurements to the community (1-day community mobilisation in each community).	3 districts in Gauteng – urban (Tshwane, Johannesburg, Ekurhuleni) and two districts in KZN – rural (Zululand, Umzinyathi)	Explore stakeholders, and participants’ experiences of MUAC intervention	Community task teams, participating LHWs and mothers/caregivers	Focus group discussions (*n* = 21)
14	^52^	Same intervention as in^40^	Rietvlei and Umlazi (KwaZulu-Natal), Paarl (Western Cape); conducted 2006	Explores mothers’ experiences of infant feeding after receiving peer counselling promoting exclusive breast or formula feeding.	Mothers (*n* = 17)	Semi-structured interviews
15	^53^	**School-readiness intervention,** part of NDoH community-based PHC outreach in crèches and day-cares. Aims to provide parents with the skills to contribute to their children’s educational and intellectual development. Includes initial assessments of the children’s readiness for school, followed by home visits to the specific children’s parents. Home visits conducted by nursing students registered for a 1-year Advanced Diploma in CHN, University of the Free State.	Heidedal, Bloemfontein, Free State intervention implemented between 2010–12	Understand the experiences of parents regarding the school-readiness intervention for preschool children facilitated by nursing students.	Parents (*n* = 24)	3 focus group discussions
16	^54^	**KZN DoH MCH programme.** LHWs allocated a number of households that they serve and are expected to visit regularly (3–5 households per day). During household visits they perform a variety of functions, incl. treatment support and home-based care, as well as MCH activities (visiting all mothers during pregnancy and in the postnatal period to provide education and support in several key areas including antenatal care attendance, planning for delivery of the baby, postnatal care and support for infant feeding).	Five communities in three districts in KZN; conducted 2014–15	Explore the performance of LHWs providing maternal and child health services at household level and the quality of the LHW-mother interaction.	15 LHWs and 30 mothers/pregnant women	LHW household visits to mothers were observed and field notes taken, followed by in-depth interviews with mothers and LHWs.

MOVIE, Mobile Video Intervention for Exclusive breastfeeding; CHN, Community Health Nursing; EBF, exclusive breastfeeding; ECSS, Eastern Cape Supervision Study; FGD, focus group discussion; IMH, infant mental health; KZN, KwaZulu-Natal; LHW, lay health worker; MCH, maternal and child health; MM, mentor mothers; MMZ, mentor mothers Zithulele; MUAC, mid-upper arm circumference; DoH, Department of Health; NDoH, National Department of Health; HCSI, Home Visit Communication Skills Inventory; RCT, randomised controlled trial; HIV, human immunodeficiency virus; AIDS, acquired immunodeficiency syndrome; PHC, primary health care; PMTCT, prevention of mother-to-child transmission.

**Table 2:** Summary of Qualitative Findings (SoQF) table.

Summary of review finding	Studies contributing to the review finding	GRADE-CERQual assessment of confidence in the evidence	Supporting data (selection)	Explanation of GRADE-CERQual assessment
**Theme 1: Facilitators of recipients’ acceptance of home visits**				
**Finding 1: Acquiring knowledge and skills.** Many mothers appreciate the educational element of home visits, whereby through education, hands-on activities, problem-solving and advice they learn new information about their own and child’s health that is relevant and understandable.	^38,42,47,51,53,54^	High confidence	‘We were taught that the porridge is made in different ways … We also learnt a lot about breastfeeding … That really inspired us as young people.’ (Mother, NDoH Family MUAC Project)^51^ ‘There are so many things that I learned. I didn’t know that you have to wash your hands before you touch a baby’s bottle, then wash the bottle and prepare food for the baby, I didn’t know that. Even feeding the child, I thought that you just feed the child … I didn’t know the motive behind it, you see … So after she came and explained to me about breastfeeding … telling me that the child must never miss the scale dates [*at the clinic*], I became alright, I saw myself as a good person and I did things the way she told me to.’ (Mother, Enable Mentor Mother Programme)^47^	
**Finding 2: Time to learn and express needs.** Many mothers and LHWs perceive home visits as enabling mothers the opportunity to have more time with a healthcare provider compared to in a clinic setting, in turn allowing for greater learning, discussion and expression of needs.	^47,50,51,54^	High confidence	‘The other thing that we like is that about these people [*CHWs*] coming to our homes is that they are patient and speak to you in a proper manner unlike the clinic, you can’t even explain at the clinic that the child was vomiting or what was happening because you are already scared. When they [*CHWs*] come to your house you can explain everything that you see with the child that you are not happy about, you understand?’ (Mother, NDoH Family MUAC Project)^51^ ‘There are so many people in the clinic … at home you arrive and relax with your patient so that the mother can freely explain the problem and you have time to help her even if you don’t have anything to give but when you go out she would be satisfied.’ (LHW, Eastern Cape Supervision Study)^50^ ‘During the [*CHW*] visits there is enough time to talk unlike the time at the clinic. I liked that because we receive more information than we get at the clinic because the time at the clinic is not enough, nurses are rushing to service everyone.’ (Mother, KZN DoH MCH programme)^54^	
**Finding 3: Psychosocial support** Many mothers value the supportive role of home visits, which they attribute to the respect, responsiveness and encouragement of LHWs and the continuity of their interactions with them.	^38,42,47,51,53,54^	High confidence	‘Our Mentor Mother is very diligent, I love her … it doesn’t matter where she is, if she is at work, she would say after work I will come and listen to what you are calling me for, here at home we like inviting her even if we just sitting we will just call her.’ (Mother, Enable Mentor Mother Programme)^47^ ‘The help I get, it’s every time when the Mentor Mother visited me she always encourage, she always give that hope that I must stay strong. I’m not alone.’ (Mother, Mentor Mothers Zithulele)^42^ ‘When I met [*my mentor*] I was always crying but she’s the one who wiped away my tears and encouraged me, telling me that, ‘You are not alone who are having [*HIV*], there are many women living with [*HIV*]’… The mentor mother plays a big role to me because by coming and visiting it makes my heart become better.’ (Mother, Mentor Mothers Zithulele)^42^ ‘… [*S*]he [*the child*] battled in the beginning, but afterwards they showed me how … I also battled and got impatient, but they encouraged me … and then I learnt with the child.’ (Mother, School-readiness intervention)^53^	
**Finding 4: Reduced clinic visits** Some mothers and LHWs perceive home visits as reducing the number of clinic visits mothers need to make and value the associated time, convenience and (opportunity) cost savings this generates for mothers.	^51^	Very low confidence	‘We don’t have a clinic around where we can take the children to, which could be a reason why our children might not be growing well. That is why we are scared to go to the clinics because even if you were to spend your last cent when you get to the clinic, you find one very long queue when you get there at 5am, you will only leave 5pm when they have already closed or at other times you wouldn’t have received assistance.’ (Mother, NDoH Family MUAC Project)^51^	Finding downgraded because of moderate concerns about methodological limitations, minor concerns about coherence (ambiguous data), serious concerns about relevance (partial and indirect relevance) and serious concerns about adequacy.
**Finding 5: Better access to, and relationships with, healthcare facilities and staff** Some mothers perceive home visits as enhancing their access to, and relationships with, healthcare facilities and staff, through the provision of referral letters or logistical information or the confidence they gain from home visits.	^47,48,51,54^	Moderate confidence	‘But if they are giving birth in a clinic or carrying something from us, they pay attention to them, they don’t queue.’ (Mother, NDoH Family MUAC Project)^51^ ‘It helped me and she was complimented at the clinic as well. They asked what brought me to the clinic. I told them it was the CHW who said I must come to the clinic. They said she has done well and I went to the clinic early during my pregnancy.’ (Mother, KZN DoH MCH programme)^54^ ‘You know, when a nurse says something, it’s hard not to know what it means. Because it’s hard to say how you will answer. Right now, when the nurse says something, I say that I have been taught about this too. Even though I don’t have a certificate like you, but I have been taught. Now my mother will be able to fight for herself.’ (Mother, NDoH Family MUAC Project)^51^	Finding downgraded because of minor concerns about methodological limitations, minor concerns about coherence (ambiguous data), and moderate concerns about adequacy.
**Finding 6: LHWs coming from same community** Many mothers prefer LHWs coming from the same community as themselves because they feel this makes them more accessible, familiar, approachable, or able to understand their experiences and context. Some LHWs similarly prefer to work with mothers from the same community as they feel this facilitates trust and relationship-building and in turn acceptance among mothers.	^41,47,49,50,51,54^	Moderate confidence	‘I get in touch with her all the time because she is someone who is lives nearby.’ (Mother, KZN DoH MCH programme)^54^ ‘I think it is better if the health worker is from the same community as you, because she will know the lives of the people in your community. It will be easy for her to help the people because they are people from her community; a person cares about their community … rather than going to a community that they don’t know anything about.’ (Community member, KZN DoH MCH programme)^41^ ‘When this program came, they started from the ground up and joined us in our homes, which means that CHW are the people we live with in society and they are the people you can talk to and they will show you.’ (Mother, NDoH Family MUAC Project)^51^ ‘I was born in [*village name*]. I also grew up there, they know me, so when I am talking they are able to ask what they do not understand … a person can say: ‘so what will happen with a certain thing?’ They speak freely because they know me.’ (LHW, Eastern Cape Supervision Study)^50^ ‘I feel very happy because I work with people that I know most of the time, that also know me, trust me and know that I am so-and-so’s child and what my home is like because people are able to be open.’ (LHW, Enable Mentor Mother Programme)^51^	Finding downgraded because of minor concerns about methodological limitations, moderate concerns about coherence (contradictory data), and minor concerns about relevance (indirect relevance).
**Finding 7: Incorporating innovative digital technologies: recipients** Incorporating digital devices containing mobile video content as health promotion and teaching tools during home visits are valued by some mothers and may enhance their acceptance of home visits more generally.	^37,39^	Very low confidence	‘I like it because it uplifts our work. It shows people how important is our work.’ (LHW, Philani Health and Nutrition Project + tablets with teaching videos)^39^	Finding downgraded because of moderate concerns about methodological limitations, minor concerns about coherence (ambiguous data), serious concerns about relevance (partial relevance) and serious concerns about adequacy.
**Finding 8: Perceived positive psychological and behavioural impact** Mothers perceive home visits to have various positive effects, including facilitating changes in behaviour, increased confidence in health-related decision-making, a sense of responsibility for their own and child’s health, or feelings of gratitude for receiving something worthwhile.	^42,47,51,53^	Moderate confidence	‘It makes me feel good when I see she takes her pen and paper … and they do good at school and the teacher asks us to tell them where they have learnt this.’ (Mother, School-readiness intervention)^53^ ‘CHWs visit homes apart from what we are doing now. They used to come to check if the child is vaccinated and to check many other things. That made us motivated because we knew that the CHW would come to check the card.’ (Mother, NDoH Family MUAC Project)^51^	Finding downgraded because of minor concerns about methodological limitations, moderate concerns about coherence (ambiguous data), minor concerns about relevance (indirect relevance) and moderate concerns about adequacy.
**Theme 2: Barriers to recipients’ and the broader community’s acceptance of home visits**				
**Finding 9: Distrust of LHWs** Some mothers and community members are less accepting of home visits because of their distrust of LHWs conducting the home visits.	^39,41,50,54^	High confidence		
**Finding 9.1: Privacy and confidentiality** Some mothers and community members distrust LHWs because of concerns related to privacy and confidentiality, including that LHWs may disclose private information to community members, or concerns about discussing confidential information during home visits when other family members are present.	^39,41,54^	Moderate confidence	‘I think they should come from a different community, not the same community as me. … Because if she is from the same community as me she may get tempted and end up telling other people [*about my secrets*].’ (Community member, KZN DoH MCH programme)^41^ ‘I know from experience that they [*CHWs*] do go around talking about other people’s problems.’ (Professional nurse, KZN DoH MCH programme)^41^ ‘I thought that they [*clients*] were going to think that you will record them and take pictures of them. I thought they were going to say that as we drink this way […], you are now going to record us and take pictures of us.’ (LHW, Philani Health and Nutrition Project + tablets with teaching videos)^39^ ‘It would be a problem if she has not told anyone [*her HIV status*] at home. When the health worker comes, she must state a reason for her visit and [*name*] can then look for a private place that they could go to so that they can talk alone, because she has not told anyone in her family about her status.’ (Community member, KZN DoH MCH programme)^41^	Finding downgraded because of minor concerns about methodological limitations, minor concerns about coherence (ambiguous data), minor concerns about relevance (partial relevance) and moderate concerns about adequacy.
**Finding 9.2: LHW Gender** Some mothers and community members distrust home visits conducted by male LHWs because they have concerns about personal safety, question whether a male could provide maternal healthcare and/or hold certain gendered norms about the role of men in antenatal and postnatal periods.	^41^	Very low confidence	‘In terms of tradition, if someone is sick at home, we are from the rural areas; we are not from the townships. If a female person is sick at home, even I as the head of the household do not touch her. It’s the women who are neighbours who will come and assist her in whatever way that she needs to be assisted. We men will stay outside. We do not even go inside the house while the sick woman is being assisted by the other women. That is the traditional way.’ (Community member, KZN DoH MCH programme)^41^	Finding downgraded because of minor concerns about coherence (ambiguous data), serious concerns about relevance (partial relevance) and serious concerns about adequacy.
**Finding 9.3: Perceived lack of competencies** Some mothers and community members distrust LHWs because they perceive them to lack the competencies of healthcare service providers. Various factors may contribute to this perception, including LHWs relationship with clinic staff, LHWs’ voluntary or temporary employment status, or LHWs having limited access to essential tools and equipment.	^41,44,50^	Moderate confidence	‘CHWs felt their credibility was challenged by PNs [*professional nurses*], who made use of their help in busy times but treated them with contempt and disrespect when not needed. These power dynamics played out in the clinics and affected the perceived competency of CHWs by the community and undermined the trust individuals place in the CHW’s ability to provide care.’ (Study author, KZN DoH MCH programme)^41^ ‘Even if we have those referral forms we still get undermined. They say we think we are doctors and they say this in front of the patient.’ (LHW, KZN DoH MCH programme)^41^ ‘We feel as if we are not welcome. … If the clinic staff do not respect what we are doing there at the clinic, then how do they expect the community to respect us? They don’t value our presence.’ (LHW, KZN DoH MCH programme)^41^ ‘CHWs also reported that some community members undermined CHWs because they saw them as voluntary workers, occupying a lower status than nurses. Although CHWs in this program earned a salary, there were discussions about the level of remuneration. Furthermore, CHWs were on temporary contracts as opposed to permanent ones.’ (Study author, Eastern Cape Supervision Study)^50^ ‘To them it is like we are not employed as compared to those who are working in the clinic so in that case we need to sit down with that person and explain to her about our job and try to show her the help we bring to the community.’ (LHW, Eastern Cape Supervision Study)^50^ ‘Having transport and all the necessary machines like BP [*blood pressure*] machines and scales because it gives us dignity and respect from the community.’ (LHW, Eastern Cape Supervision Study)^44^	Finding downgraded because of minor concerns about coherence (ambiguous data), moderate concerns about relevance (partial relevance) and moderate concerns about adequacy.
**Finding 10: Stigma associated with home visits** Some mothers and community members are less accepting of home visits because of the stigma associated with them, including the belief that they are only for people living with HIV/AIDS or fear of being judged as weak for needing support.	^38,39^	Moderate confidence	‘Most of the time my sister, they do not know what is it that we do, they do not know our work. They tell themselves that we visit people that are HIV positive.’ (LHW, Philani Health and Nutrition Project + tablets with teaching videos)^39^ ‘It is hard for the mom just to say … I need help … you see, we are brought up in this kind of families that … you are a woman, stand for yourself, do this and do this, the right way.’ (LHW, Ububele Mother-Baby Home Visiting project)^38^	Finding downgraded because of minor concerns about methodological limitations, minor concerns about coherence (ambiguous data), moderate concerns about relevance (partial relevance) and moderate concerns about adequacy.
**Theme 3: Facilitators of lay health workers’ acceptance of home visits**				
**Finding 11: Empowering, validating, employment and convenience** LHWs may be generally supportive of home visits because of feeling a sense of empowerment, dignity, purpose, and strength because of their role, valued for making a difference, pride in earning a salary or appreciative of the convenience of their job.	^42,49,51^	Moderate confidence	‘… encourage us in our work, and that we do our work faithfully so that I will also be proud of reporting on the work that I have done.’ (LHW, NdoH Family MUAC Project)^51^ ‘that thing of being called nurses … that means there is a big role you play.’ (LHW, Enable Mentor Mother Programme)^49^ ‘And after ten visits, you can see that I made an impact, even if I didn’t give them money or whatever, but the mother feels better ….’ (LHW, Ububele Mother-Baby Home Visiting project)^38^ ‘[*E*]ven if the money is little, that hope of having money at month-end, it can make you feel confident even when you walk on the road.’ (LHW, Enable Mentor Mother Programme)^49^ ‘Our goal is that the babies must grow with that healthy body … As we visit that woman who is pregnant and living with HIV, we will advise her to go to the clinic so that she can take treatment. The more that they will give birth, the more they will give birth to that baby who is negative. So we make less the population who has HIV.’ (LHW, Mentor Mothers Zithulele)^42^	Finding downgraded because of minor concerns about methodological limitations, moderate concerns about coherence (ambiguous data), and moderate concerns about adequacy.
**Finding 12: Incorporating innovative digital technologies: LHWs** Incorporating digital devices containing mobile video content as health promotion and teaching tools during home visits may be valued by LHWs and may enhance their acceptance of home visits more generally.	^37,39^	Very low confidence	‘I do not want to lie, I became very proud, I saw that it is now that I am working, I saw my dignity because there is that thing [*the tablet*] […].’ (CHW, Philani Health and Nutrition Project + tablets with teaching videos)^39^ ‘You enter a house and you would open a folder and we also have the household assessment forms, you have to do it and I think it will minimise the time that you spend in one house.’ (LHW, Philani Health and Nutrition Project + tablets with teaching videos)^39^	Finding downgraded because of moderate concerns about methodological limitations, minor concerns about coherence (ambiguous data), serious concerns about relevance (partial relevance) and serious concerns about adequacy.
**Theme 4: Barriers to lay health workers’ acceptance of home visits and the feasibility of home visits**				
**Finding 13: Boundaries and emotional burdens** Many LHWs find it difficult to maintain boundaries with home visiting clients and to balance professional and personal obligations, which can have a negative impact on their emotional well-being.	^38,40,49^	High confidence	‘It’s painful, because I know how to starve and I started to think about when I was young, living with my sister at Limpopo, starving, no food … I started to think about myself when I was eating food of dogs at the neighbours, and this is the mother I’m visiting, she’s breastfeeding, she’s hungry, she has to eat so that she can breastfeed the baby, and what must I do now? Am I going to sit and say, I can feel your pain? I can’t, I’m a human being. I can’t just say “ja, it’s difficult.”’ (LHW, Ububele Mother-Baby Home Visiting project)^38^ ‘At home they now know, I just go to sleep when there is something troubling me. My daughter would ask me, “mom, what happened in the field? why do you come home troubled?” I would tell her that, “no, stop, I just need to sleep first,” I would then sleep. When I wake up, [*I*] tell them that it is because we work with people and sometimes the problems would be too much in the community.’ (LHW, Enable Mentor Mother Programme)^49^	
**Finding 14: Training, supervision, and support** LHWs of home visiting programmes have many gaps in the necessary knowledge and skills because of inadequate training, supervision, and support. These deficits make it difficult for LHWs to perform their tasks and may in turn undermine programme credibility and community acceptance. Increased training, supervision and support may positively impact on LHWs’ ability to carry out their work because of the increased knowledge, confidence, motivation and sense of accountability it may generate.	^40,41,43,44,49,50,51,54^	High confidence	‘I do have knowledge but it is not adequate. Perhaps I need to be given additional information. There are questions that they ask where you find that I will not be confident when I respond to them.’ (LHW, KZN DoH MCH programme)^54^ ‘We discovered that there were things that they didn’t properly understand like the virus in the milk, some said yes there is some said no.’ (Senior researcher, Promise EBF study)^40^ ‘There is nobody knowing that you are going into the field and actually seeing people, there is no checking up if you … don’t create systems where people know that they will be checked upon, some people will abuse it. The second thing is that the support is also really poor, people feel that they are isolated, on their own, there is nobody who can give them advice, there is nobody who can tell them where the patient should go and that is what is so useful to have a link into the hospital.’ (Programme manager, Eastern Cape Supervision Study)^43^ ‘She [*clinic-based team leader*] would say that she will never go to my village because it is far, she never supervised me even for one day.’ (Mother, Eastern Cape Supervision Study)^50^ ‘She [*LHW supervisor*] had never gone to the field with me … you find that you do not get assistance with certain things that you need to be assisted in when you visit homes. You end up having to wait for the next meeting at the clinic, and that is the only time you can ask about things that were challenging to you when you were trying to educate the family.’ (LHW, KZN DoH MCH programme)^54^ ‘The Philani training made a huge difference in my work experience because it had materials; we were trained and received the materials, you get trained then you also do what you were trained for, and clients notice that there’s a huge difference.’ (LHW, Eastern Cape Supervision Study)^44^ ‘It’s also beneficial to us because I gained a lot of knowledge and understood my work more and it becomes easier as you have a supervisor checking up on you.’ (LHW, Eastern Cape Supervision Study)^44^ ‘I feel important as a worker that my boss comes to check the work I do.’ (LHW, NDoH Family MUAC Project)^51^ ‘Would the way we worked be the same if we went out on our own instead of being monitored? No, it wouldn’t be the same.’ (LHW, NDoH Family MUAC Project)^51^ ‘Telephone call support it’s not effective at all for myself because the peer supporter only tells you what she thinks you need to know but you haven’t seen what she did and that’s the difference. But when you’re there you are able really to give the support that she needs because you’ve seen what she was doing and you see what she needed to do and you also see where she can improve what she could have done.’ (LHW supervisor, Promise EBF study)^40^	
**Finding 15: Practical and logistical challenges** Many LHWS face various practical and logistical challenges when conducting home visits, including inadequate transportation and essential tools and equipment, mobility of clients, and personal safety issues. These challenges make it difficult for LHWs to perform their tasks and may in turn undermine programme credibility and community acceptance.	^39,40,43,44,50,51,54^	High confidence	‘It’s [*exact hours removed for* *de-identification*] hours walking … and there is no transportation … it becomes so painful but you don’t have a choice because you have to go to work or you have to visit that house.’ (LHW, Eastern Cape Supervision Study)^50^ ‘One of the biggest challenges is that they relocate from where they are staying in [*Place*] because they don’t permanently stay in these areas, during follow-up we are told that the person no longer stays there.’ (LHW, NDoH Family MUAC Project)^51^ ‘The areas are not safe for peer supporters … we had a peer supporter who went visiting the house and somebody was shot … in her presence ….’ (LHW Supervisor, Promise EBF study)^40^ ‘Those villages are far from each other and … to get to other village you have to pass through the forest and that is not easy for ladies to pass through the forest because there is rape, phones are being robbed … so it won’t be easy.’ (LHW, Eastern Cape Supervision Study)^50^ ‘I was also afraid because of the places that I go to. The places that I go to criminals will be looking at me.’ (LHW, Philani Health and Nutrition Project + tablets with teaching videos)^39^ ‘My community health workers don’t have the equipment to work now, even if they go to the households they would wish to take weight of clients and wish to do that and that and they cannot do those things.’ (Operational manager, Eastern Cape Supervision Study)^43^ ‘I think that is also a problem because when we visit a household we do not have tools of trade.’ (LHW, KZN DoH MCH programme)^54^	
**Finding 16: Human resource-related issues** Human resource-related issues, including poor salaries, non-permanent contracts and increased workloads, may contribute to the high turnover and attrition rates amongst LHWs in home visiting programmes.	^40,43,50,51^	Moderate confidence	‘To them it is like we are not employed as compared to those who are working in the clinic so in that case we need to sit down with that person and explain to her about our job and try to show her the help we bring to the community.’ (LHW, Eastern Cape Supervision Study).^50^ ‘Currently the community healthcare workers … are not permanently employed, they … are uncertain of their employment and once you have job dissatisfaction you don’t get motivated or become productive because you don’t know where you fall under.’ (Operational clinic manager, Eastern Cape Supervision Study)^43^ ‘I’m still experiencing the Department of Health threatening to take these people, promising them … ‘Ah we are going to offer you something, we want you to go for homebased care training which after that we will give you salary of 3000’ [*ZAR*]’. And then I ended up losing those people.’ (LHW Supervisor, Promise EBF study)^40^	Finding downgraded because of minor concerns about methodological limitations, minor concerns about coherence (ambiguous data), and moderate concerns about adequacy.

CHWs, community health worker; DoH, Department of Health; HIV, human immunodeficiency virus; LHW, lay health worker; MCH, maternal and child health; MUAC, mid-upper arm circumference; NDoH, National Department of Health.

**Table 3:** Policy and practice implementation consideration for lay health worker home visiting programmes for families with preterm and low birthweight infants in South Africa.

**1. Living and working in the same community**	When selecting LHWs, have you considered how they will be perceived by members of the community in which they will work, for instance in terms of their sociocultural or economic background or gender? For example, if male LHWs are included have you considered whether gender norms and safety issues may reduce mother’s trust of them? Do LHWs have ways of managing relationships with recipients and creating boundaries between work and personal lives when working and living in the same community? Are there routines and standards in place to ensure that LHWs do not share recipients’ personal information with others in the community, including if digital technologies (e.g., voice or video recorders) are used? Are LHWs and community members aware of these standards? LHWs working and living in the same community may be especially vulnerable to blame in instances of incidental death, disease, or other problems during care. Have you considered how to offer help in these circumstances, for instance by providing visible support from the health system, or regular supervision and counselling? Have you considered how to foster community engagement and collaboration to help develop an enabling environment for LHWs? For example, how could the planning and implementation be done in a participatory approach with community members and community structures or organisations, for example, governmental, faith-or traditional-based, non-governmental, or civil society groups? Have you considered which community leaders or group have authority and respect in the community and in turn may be best placed to collaborate with? Or have you considered establishing appropriate bodies such as community health committees or forums to enable collaboration between community members and other role players?
**2. Work activities**	Are LHWs providing services that they see as relevant to the challenges they meet during their working day and in their interactions with community members? Have you provided LHWs with sufficient means of transportation, where necessary? Have you provided LHWs with the essential medical tools and equipment required for them to perform their tasks? Have you considered whether it might be possible for LHWs to incorporate digital devices as part of their health promotion and teaching tools? Have you put systems in place to ensure LHWs personal safety, for instance during travel or when visiting homes or neighbourhoods? Have you considered whether there is a reasonable balance between LHWs workloads and accumulation of new tasks? Have you considered whether mothers can discuss confidential information with LHWs without family members being present, and if not, how mothers’ privacy could be enhanced? Do cultural or social norms exist (e.g., gender) that could prevent some LHWs from mobilising within their community to fulfil their responsibilities? Have you considered whether community members are aware of the range of activities being performed by LHW programmes, and if not, how this awareness may be enhanced? For example, home visits may be perceived as providing only HIV/AIDS care or with a lack of knowledge regarding the broader range of functions they provide. Have you considered mobility of mothers in the area in which the programme is being implemented, and if so, how this could be accommodated?
**3. Working with other healthcare providers**	Have you considered how to ensure good working relationships between LHWs and other healthcare providers in primary care facilities (e.g., nurses or mid-level practitioners such as clinical officers, physician assistants, clinical associates)? For instance, are other healthcare providers encouraged to be respectful and responsive towards LHWs, to recognise their competencies and to accept their referrals? Has the use of LHWs added to the workload of other healthcare providers, as a result of additional tasks such as evaluation and supervision? Or do other healthcare providers perceive LHWs as lessening their workloads, and bringing complementary skills, knowledge, and experience?
**4. Referral systems**	Where LHWs are trained to refer patients with complications, are there sufficient health professionals to care for these patients? Are these health professionals willing and able to cooperate with LHWs when they receive these referrals? When referrals are necessary, do LHWs have access to functional phones to make this referral, means of transport for the patient, and funds to pay for this transport?
**5. Payment, incentives, and out-of-pocket expenses**	Are you offering LHWs sufficient salaries for their time and effort? Have you considered whether, and if so how, more secure and full-time contractual arrangements could be put in place for LHWs? Might it be possible for LHWs to receive benefits, incentives and/or opportunities for career progression? What other factors could be incorporated that could make a positive impact on the retention of LHWs? Is there a shared understanding between LHWs, programme managers and policy makers regarding how potential incentive systems reflects different tasks, different levels of responsibility or changes in skills because of further training? Have you provided LHWs with the necessary ‘work tools’, such as uniforms, mobile phones or identification (ID) badges? Have you ensured that they are reimbursed for out-of-pocket expenses? Are there systems in place whereby LHWs can voice their individual or collective concerns or complaints about incentives or other issues?
**6. Training, supervision and support**	Have you clarified what essential competencies LWHs require to fulfil their roles and perform the tasks they are required to perform? Are you offering LHWs sufficient training and supervision for them to fulfil these roles and tasks? This includes training in communication and health promotion. These tasks are often central to the LHW role but are often neglected during training. It also includes ongoing and refresher training as opposed to once-off training. Have you ensured proper systems of supervision? Do supervisors have enough skills, sufficient time, and means of transportation to provide in-field supervision in addition to more remote supervision, for example, at clinics or on the phone. Do supervisors have a good understanding of LHWs working conditions and personal circumstances? Do they provide emotional, technical and clinical support on an ongoing basis? Do LHWs have the opportunity to share their experiences with other LHWs?
**7. Community Oriented Primary Care (COPC) and integration with other community-based care**	What community-based care is already present in the target area(s)? Which principles of COPC are present in this community-based care? For example, do they include a defined community, and if so, in what terms? Do they incorporate a multidisciplinary team, comprehensive and/or equitable approach to service delivery? Are they informed by analysis and prioritisation of local health needs and resources? Do they foster community engagement and participation, and if so, which community members or structure do they collaborate with? What other values or principles might inform existing community-oriented care? How does the existing community-oriented care respond to the previous questions and prompts in this table (#1–6 above?) How can existing LHW home visit programme/interventions for families with preterm and LBW infants strengthen and develop COPC? For example, have you considered how a multidisciplinary team of HCWs could be involved, for example, nurses, doctors along with LHWs? Have you considered how a comprehensive approach to care could be fostered, incorporating aspects of health promotion, disease prevention, treatment and rehabilitation? How can the LHW home visit programme/intervention for families with preterm and LBW infants collaborate with exiting community-oriented care activities and programmes to create synergy? For example, how could services across programmes be integrated or co-ordinated to enable person-centred and continuity of care over time in a continuous process?
**8. Governance and financing**	Have you ensured there is a clear directive from provincial governments? Have you considered how ongoing political commitment by relevant stakeholders and collaboration between different levels of government can be strengthened and developed? How might a sense of ownership amongst managers at local levels and decentralised decision-making be strengthened and developed? Have you considered how adequate resources will be allocated and ongoing financial commitment sustained?
**9. Monitoring, evaluation, data and health information**	Have you ensured systems are in place to monitor implementation and identify and respond to (changing) needs and effects? What mechanisms are there for checking and verifying this information to ensure the quality of this data? How might these monitoring and evaluation systems be integrated with existing primary health care information systems and data collection processes (e.g., from households, facilities, research and other sources)?

LHW, lay health worker.

## Data Availability

The authors confirm that the data supporting the findings of this study are available within the article and Online Appendix 1, Online Appendix 2 and Online Appendix 3.
