# Supplementary material for: Home visits for preterm/low birthweight infants in South Africa: Qualitative evidence synthesis
Source: Afr J Prim Health Care Fam Med. Author manuscript; Available in PMC 2024 Dec 5. (PMC7616882; doi:10.4102/phcfm.v16i1.4701)
Supplement: Supplementary file 1 [file EMS200333-supplement-Appendices.zip › document-3.pdf]

Note: This is Online Appendix 3 of Cooper S, Kallon II, Mabetha D, et al. Home visits for preterm/low birthweight infants in South Africa: Qualitative evidence synthesis. Afr J Prm Health Care Fam Med. 2024;16(1), a4701. https://doi.org/10.4102/phcfm.v16i1.4701

Supplementary material 3: Methodological strengths and limitations of included studies

**Study Reference**	**Were the settings and context described adequately?**	**Was the sampling strategy described, and was this appropriate?**	**Was the data collection strategy described and was this appropriate?**	**Was the data analysis described, and was this appropriate?**	**Were the claims made/findings supported by sufficient evidence?**	**Was there evidence of reflexivity?**	**Did the study demonstrate sensitivity to ethical concerns?**	**Any other concerns?**
(36)	Yes	Partly	Yes	Partly	No	No	Yes	Yes
(37)	No	Yes	Yes	Yes	Yes	Yes	Partly	No
(38)	Yes	Yes	Yes	Yes	Yes	No	Yes	No
(39)	Yes	Yes	Yes	Yes	Yes	Yes	Yes	No
(40)	Yes	Yes	Yes	Yes	Yes	Partly	Yes	No
(41)	Yes	Partly	Yes	Yes	Yes	No	Yes	No
(42)	Yes	Yes	Yes	Yes	Yes	Yes	Yes	No
(43)	Yes	Yes	Yes	Yes	Yes	Partly	Yes	No
(46, 47)	Yes	Yes	Yes	Yes	Yes	No	Yes	No
(44, 45)	Yes	Yes	Yes	Yes	Yes	Yes	Yes	No
(48)	Yes	Yes	Yes	Yes	Yes	Yes	Yes	No
(49)	Yes	Yes	Yes	Yes	Yes	No	Yes	No
(50)	Yes	Partly	Partly	No	Yes	No	Yes	No
(51)	Yes	Yes	Yes	Yes	Yes	No	Yes	No
(52)	Partly	Partly	Yes	Partly	Yes	Yes	Yes	No
(53)	Partly	Yes	Yes	Yes	Yes	Yes	Yes	No
