# Supplementary material for: Home visits for preterm/low birthweight infants in South Africa: Qualitative evidence synthesis
Source: Afr J Prim Health Care Fam Med. Author manuscript; Available in PMC 2024 Dec 5. (PMC7616882; doi:10.4102/phcfm.v16i1.4701)
Supplement: Supplementary file 1 [file EMS200333-supplement-Appendices.zip › document-2.pdf]

Note: This is Online Appendix 2 of Cooper S, Kallon II, Mabetha D, et al. Home visits for preterm/low birthweight infants in South Africa: Qualitative evidence synthesis. Afr J Prm Health Care Fam Med. 2024;16(1), a4701. https://doi.org/10.4102/phcfm.v16i1.4701

Supplementary material 2: Evidence profiles

**Finding 1: Acquiring knowledge and skills.** Many mothers appreciate the educational element of home visits, whereby through education, hands-on activities, problem-solving and advice they learn new information about their own and child's health that is relevant and understandable.	
**Assessment for each GRADE-CERQual component**	
Methodological limitations	Minor concerns due to poor or partial reporting of the setting, sampling approach and data analysis and collection methods in some studies, as well as limited evidence of reflexivity in many of the studies.
Coherence	No or very minor concerns (the finding describes the key patterns in the data and is well supported by details in the contributing studies).
Relevance	No or very minor concerns regarding relevance (studies include a sufficient range of participants, settings in South Africa and interventions or programmes). While no studies focused on the interventions for pre-term or LBW infants specifically (indirect evidence) we do not anticipate there to be significant differences between pre-term/LBW infants and infants that are not pre-term/LBW that would reduce our confidence in relation to this finding.
Adequacy	No or very minor concerns (6 studies contributing a sufficient amount of data)
**Overall GRADE-CERQual assessment and explanation**	
High confidence	
**Contributing studies**	
(37, 41, 46, 50, 52, 53)	
**Finding 2: Time to learn and express needs.** Many mothers and LHWs perceive home visits as enabling mothers the opportunity to have more time with a healthcare provider compared to in a clinic setting, in turn allowing for greater learning, discussion, and expression of needs.	
**Assessment for each GRADE-CERQual component**	
Methodological limitations	Minor concerns due to poor or partial reporting of the setting, sampling approach and data analysis and collection methods in some studies, as well as limited evidence of reflexivity in many of the studies
Coherence	No or very minor concerns (the finding describes the key patterns in the data and is well supported by details in the contributing studies).
Relevance	No or very minor concerns regarding relevance (studies include a sufficient range of participants, settings in South Africa and interventions or programmes). While no studies focused on the interventions for pre-term or LBW infants specifically (indirect evidence) we do not anticipate there to be significant differences between pre-term/LBW infants and infants that are not pre-term/LBW that would reduce our confidence in relation to this finding.
Adequacy	No or very minor concerns (4 studies contributing a sufficient amount of data)
**Overall GRADE-CERQual assessment and explanation**	
High confidence	
**Contributing studies**	
(46, 49, 50, 53)	
**Finding 3: Psychosocial support**	

Many mothers value the supportive role of home visits, which they attribute to the respect, responsiveness, and encouragement of LHWs and the continuity of their interactions with them.	
**Assessment for each GRADE-CERQual component**	
Methodological limitations	Minor concerns due to poor or partial reporting of the setting, sampling approach and data analysis method in some studies, as well as limited evidence of reflexivity in many of the studies.
Coherence	No or very minor concerns (the finding describes the key patterns in the data and is well supported by details in the contributing studies).
Relevance	No or very minor concerns regarding relevance (studies include a sufficient range of participants, settings in South Africa and interventions or programmes). While no studies focused on the interventions for pre-term or LBW infants specifically (indirect evidence) we do not anticipate there to be significant differences between pre-term/LBW infants and infants that are not pre-term/LBW that would reduce our confidence in relation to this finding.
Adequacy	No or very minor concerns (6 studies contributing sufficient amount of data)
**Overall GRADE-CERQual assessment and explanation**	
High confidence	
**Contributing studies**	
(37, 41, 46, 50, 52, 53)	
**Finding 4: Reduced clinic visits**<br>Some mothers and LHWs perceive home visits as reducing the number of clinic visits mothers need to make, and value the associated time, convenience and (opportunity) cost savings this generates for mothers.	
**Assessment for each GRADE-CERQual component**	
Methodological limitations	Moderate concerns due to partial reporting of the sampling and data collection methods, no reporting of the data analysis methods and no evidence of reflexivity.
Coherence	Minor concerns because there is insufficient data to support all aspects of the review finding (e.g. relatively thin data on the association) (ambiguous data)
Relevance	Serious concerns because evidence from only 1 study exploring 1 programme in the country contributed to this finding (partial relevance). We also anticipate there may be differences between pre-term/LBW infants and infants that are not pre-term/LBW in relation to the value of reduced clinic visits, which may reduce our confidence in relation to this finding (indirect relevance).
Adequacy	Serious concerns because only 1 study with relatively limited data contributed to this finding.
**Overall GRADE-CERQual assessment and explanation**	
Very low confidence	Finding downgraded because of moderate concerns about methodological limitations, minor concerns about coherence (ambiguous data), serious concerns about relevance (partial and indirect relevance) and serious concerns about adequacy.
**Contributing studies**	
(50)	
**Finding 5: Better access to, and relationships with, healthcare facilities and staff**<br>Some mothers perceived home visits as enhancing their access to, and relationships with, healthcare facilities and staff, through the provision of referral letters or logistical information or the confidence they gain from home visits.	
**Assessment for each GRADE-CERQual component**	
Methodological limitations	Minor concerns due to poor or partial reporting of the setting, sampling approach and data collection and analysis methods in 1 study, as well as limited evidence of reflexivity in many of the studies
Coherence	Minor concerns because there is insufficient data to support all aspects of the review finding (e.g. relatively thin data on logical information and the confidence gained) (ambiguous data)

Relevance	No or very minor concerns regarding relevance (studies include a sufficient range of participants, settings in South Africa and interventions or programmes). While no studies focused on the interventions for pre-term or LBW infants specifically (indirect evidence) we do not anticipate there to be significant differences between pre-term/LBW infants and infants that are not pre-term/LBW that would reduce our confidence in relation to this finding.
Adequacy	Moderate concerns (4 studies contributing relatively thin data to this finding)
**Overall GRADE-CERQual assessment and explanation**	
Moderate confidence	Finding downgraded because of minor concerns about methodological limitations, minor concerns about coherence (ambiguous data), and moderate concerns about adequacy.
**Contributing studies**	
(46, 47, 50, 53)	
**Finding 6: LHWs coming from same community**<br>Many mothers prefer LHWs coming from the same community as themselves because they feel this makes them more accessible, familiar, approachable, or able to understand their experiences and context. Some LHWs similarly prefer to work with mothers from the same community as they feel this facilitates trust and relationship-building and in turn acceptance amongst mothers.	
**Assessment for each GRADE-CERQual component**	
Methodological limitations	Minor concerns due to poor or partial reporting of the setting, sampling approach and data collection and analysis methods in 1 study, as well as limited evidence of reflexivity in many of the studies.
Coherence	Moderate concerns because there was contradictory data that did not fit the interpretation in this finding (some LHWs and mothers did not prefer LHWs coming from the same community and various concerns were highlighted by the studies regarding LHWs coming from the same community as mothers) (contradictory data).
Relevance	Minor concerns regarding relevance (studies include a sufficient range of participants, settings in South Africa and interventions or programmes. However, we anticipate there may be differences between pre-term/LBW infants and infants that are not pre-term/LBW in relation to the contradictory data (the former may have more concerns about LHWs coming from the same community due to potential stigma associated with pre-term/LBW infants and associated concerns of privacy and confidentiality), which may reduce our confidence in relation to this finding (indirect relevance).
Adequacy	No or very minor concerns (6 studies contributing sufficient amount of data)
**Overall GRADE-CERQual assessment and explanation**	
Moderate confidence	Finding downgraded because of minor concerns about methodological limitations, moderate concerns about coherence (contradictory data), and minor concerns about relevance (indirect relevance).
**Contributing studies**	
(40, 46, 48-50, 53)	
**Finding 7: Incorporating innovative digital technologies: recipients**<br>Incorporating digital devices containing mobile video content as health promotion and teaching tools during home visits are valued by some mothers and may enhance their acceptance of home visits more generally.	
**Assessment for each GRADE-CERQual component**	
Methodological limitations	Moderate concerns because 1 study had poor or partial reporting of the sampling and data collection methods and insufficient data to support the claims made, and both studies revealed no evidence of reflexivity.
Coherence	Minor concerns because data supporting the link between valuing digital technologies and enhancing acceptance of home visits more generally was not sufficiently described in the studies (ambiguous data).
Relevance	Serious concerns because the evidence contributing to this finding comes from 2 studies focused on 1 programme (Philani) in 1 urban setting (Khayelitsha) in the country (partial relevance).

Adequacy	Serious concerns because only 2 studies with relatively limited data contributed to this finding.
**Overall GRADE-CERQual assessment and explanation**	
Very low confidence	Finding downgraded because of moderate concerns about methodological limitations, minor concerns about coherence (ambiguous data), serious concerns about relevance (partial relevance) and serious concerns about adequacy.
**Contributing studies**	
(36, 38)	
**Finding 8: Positive impact** Mothers perceive home visits to have various positive effects, including facilitating changes in behaviour, increased confidence in health-related decision-making, a sense of responsibility for their own and child's health, or feelings of gratitude for receiving something worthwhile.	
**Assessment for each GRADE-CERQual component**	
Methodological limitations	Minor concerns due to poor or partial reporting of the setting, sampling approach and data collection and analysis methods in some studies, as well as limited evidence of reflexivity in many of the studies.
Coherence	Moderate concerns because not all the positive effects identified were clearly or sufficiently described (e.g. behaviour change; increased confidence) (ambiguous data)
Relevance	Minor concerns regarding relevance (studies include a sufficient range of participants, settings in South Africa and interventions or programmes. However, we anticipate there may be differences between pre-term/LBW infants and infants that are not pre-term/LBW- the former may require more complicated and intensive care needs which may reduce the positive impacts of LHW home visits and in turn our confidence in relation to this finding (indirect relevance).
Adequacy	Moderate concerns (4 studies contributing relatively thin data)
**Overall GRADE-CERQual assessment and explanation**	
Moderate	Finding downgraded because of minor concerns about methodological limitations, moderate concerns about coherence (ambiguous data), minor concerns about relevance (indirect relevance) and moderate concerns about adequacy.
**Contributing studies**	
(41, 46, 50, 52)	
**Finding 9: Distrust of LHWs** Some mothers and community members are less accepting of home visits because of their distrust of LHWs conducting the home visits	
**Assessment for each GRADE-CERQual component**	
Methodological limitations	Minor concerns due to poor or partial reporting of the setting in 1 study and limited evidence of reflexivity in many of the studies.
Coherence	No or very minor concerns (the finding describes the key patterns in the data and is well supported by details in the contributing studies).
Relevance	No or very minor concerns regarding relevance (studies include a sufficient range of participants, settings in South Africa and interventions or programmes). While no studies focused on the interventions for pre-term or LBW infants specifically (indirect evidence) we do not anticipate there to be significant differences between pre-term/LBW infants and infants that are not pre-term/LBW that would reduce our confidence in relation to this finding.
Adequacy	No or very minor concerns (4 studies contributing sufficient amount of data)
**Overall GRADE-CERQual assessment and explanation**	
High confidence	
**Contributing studies**	

(38, 40, 49, 53)	
**Finding 9.1: Privacy and confidentiality**<br>Some mothers and community members distrust LHWs because of concerns related to privacy and confidentiality, including that LHWs may disclose private information to community members or concerns about discussing confidential information during home visits when other family members are present.	
**Assessment for each GRADE-CERQual component**	
Methodological limitations	Minor concerns due to poor or partial reporting of the setting in 1 study and limited evidence of reflexivity in 2 of the studies.
Coherence	Minor concerns because one aspect of the finding was not sufficiently described in the studies (discussing confidential information at home) (ambiguous data)
Relevance	Minor concerns regarding relevance (studies are focused on only 2 programmes in 2 settings in the country- partial relevance. While no studies focused on the interventions for pre-term or LBW infants specifically (indirect evidence) we do not anticipate there to be significant differences between pre-term/LBW infants and infants that are not pre-term/LBW that would reduce our confidence in relation to this finding).
Adequacy	Moderate concerns (3 studies contributing relatively thin amounts of data)
**Overall GRADE-CERQual assessment and explanation**	
Moderate confidence	Finding downgraded because of minor concerns about methodological limitations, minor concerns about coherence (ambiguous data), minor concerns about relevance (partial relevance) and moderate concerns about adequacy.
**Contributing studies**	
(38, 40, 53)	
**Finding 9.2: LHW Gender**<br>Some mothers and community members distrust home visits conducted by male LHWs because they have concerns about personal safety, question whether a male could provide maternal healthcare and/or hold certain gendered norms about the role of men in antenatal and postnatal periods.	
**Assessment for each GRADE-CERQual component**	
Methodological limitations	No or very minor concerns.
Coherence	Minor concerns because some aspects of the finding were not clearly or sufficiently described (question whether a male could provide maternal healthcare and gendered norms about the role of men in antenatal and postnatal periods) (ambiguous data)
Relevance	Serious concerns because the evidence contributing to this finding comes from 1 study focused on 1 programme in 1 urban setting in the country (partial relevance).
Adequacy	Serious concerns because only 1 study with relatively limited data contributed to this finding
**Overall GRADE-CERQual assessment and explanation**	
Very low confidence	Finding downgraded because of minor concerns about coherence (ambiguous data), serious concerns about relevance (partial relevance) and serious concerns about adequacy.
**Contributing studies**	
(40)	
**Finding 9.3: Perceived lack of competencies**<br>Some mothers and community members distrust LHWs because they perceive them to lack the competencies of healthcare service providers. Various factors may contribute to this perception, including LHWs relationship with clinic staff, LHWs' voluntary or temporary employment status, or LHWs having limited access to essential tools and equipment.	
**Assessment for each GRADE-CERQual component**	

Methodological limitations	No or very minor concerns.
Coherence	Minor concerns because one aspect of the finding was not clearly or sufficiently described (LHWs' voluntary or temporary employment status) (ambiguous data)
Relevance	Moderate concerns because the evidence contributing to this finding comes from 2 studies both conducted in rural settings in South Africa and focused on only 2 programmes (partial relevance). While no studies focused on the interventions for pre-term or LBW infants specifically (indirect evidence) we do not anticipate there to be significant differences between pre-term/LBW infants and infants that are not pre-term/LBW that would reduce our confidence in relation to this finding).
Adequacy	Moderate concerns (2 studies contributing relatively thin data)
**Overall GRADE-CERQual assessment and explanation**	
Moderate confidence	Finding downgraded because of minor concerns about coherence (ambiguous data), moderate concerns about relevance (partial relevance) and moderate concerns about adequacy.
**Contributing studies**	
(41, 44, 50)	
**Finding 10: Stigma associated with home visits**<br>Some mothers and community members are less accepting of home visits because of the stigma associated with them, including the belief that they are only for people living with HIV/AIDS or fear of being judged as weak for needing support.	
**Assessment for each GRADE-CERQual component**	
Methodological limitations	Minor concerns due to poor or partial reporting of the setting in 1 study and limited evidence of reflexivity and sensitivity to ethical concerns in 1 study.
Coherence	Minor concerns because one aspect of the finding was not clearly or sufficiently described (fear of being judged as weak for needing support) (ambiguous data)
Relevance	Moderate concerns because the evidence contributing to this finding comes from 2 studies both conducted in urban settings (partial relevance) and focused on only 2 programmes. While no studies focused on the interventions for pre-term or LBW infants specifically (indirect evidence) we do not anticipate there to be significant differences between pre-term/LBW infants and infants that are not pre-term/LBW that would reduce our confidence in relation to this finding).
Adequacy	Moderate concerns (2 studies contributing relatively thin data)
**Overall GRADE-CERQual assessment and explanation**	
Moderate confidence	Finding downgraded because of minor concerns about methodological limitations, minor concerns about coherence (ambiguous data), moderate concerns about relevance (partial relevance) and moderate concerns about adequacy.
**Contributing studies**	
(37, 38)	
**Finding 11: Empowering, validating, employment and convenience**<br>LHWs may be generally supportive of home visits due to feeling a sense of empowerment, dignity, purpose and strength because of their role, valued for making a difference, pride in earning a salary or appreciative of the convenience of their job.	
**Assessment for each GRADE-CERQual component**	
Methodological limitations	Minor concerns due to poor or partial reporting of the setting and data collection and analysis methods in some studies and limited evidence of reflexivity in 3 of the studies.
Coherence	Moderate concerns because the specific factors highlighted in the review findings as contributing to LHW support of home visits were insufficiently described or unclear (ambiguous data)
Relevance	No or very minor concerns regarding relevance (studies include a sufficient range of participants, settings in South Africa and interventions or programmes). While no studies focused on the interventions for pre-term or LBW infants specifically (indirect evidence) we do not anticipate there to

	be significant differences between pre-term/LBW infants and infants that are not pre-term/LBW that would reduce our confidence in relation to this finding.
Adequacy	Moderate concerns (4 studies contributing relatively thin data)
**Overall GRADE-CERQual assessment and explanation**	
Moderate confidence	Finding downgraded because of minor concerns about methodological limitations, moderate concerns about coherence (ambiguous data), and moderate concerns about adequacy.
**Contributing studies**	
(41, 48-50)	
**Finding 12: Incorporating innovative digital technologies: LHWs**<br>Incorporating digital devices containing mobile video content as health promotion and teaching tools during home visits may be valued by LHWs and may enhance their acceptance of home visits more generally.	
**Assessment for each GRADE-CERQual component**	
Methodological limitations	Moderate concerns because 1 study had poor or partial reporting of the sampling and data collection methods and insufficient data to support the claims being made, and both studies revealed no evidence of reflexivity.
Coherence	Minor concerns because data supporting the link between valuing digital technologies and enhancing acceptance of home visits more generally was not described in great detail by the studies (ambiguous data).
Relevance	Serious concerns because the evidence contributing to this finding comes from 2 studies focused on 1 programme (Philani) in 1 urban setting (Khayelitsha) in the country (partial relevance).
Adequacy	Serious concerns because only 2 studies with relatively limited data contributed to this finding.
**Overall GRADE-CERQual assessment and explanation**	
Very low confidence	Finding downgraded because of moderate concerns about methodological limitations, minor concerns about coherence (ambiguous data), serious concerns about relevance (partial relevance) and serious concerns about adequacy.
**Contributing studies**	
(36, 38)	
**Finding 13: Boundaries and emotional burdens**<br>Many LHWs find it difficult to maintain boundaries with home visiting clients and to balance professional and personal obligations, which can have a negative impact on their emotional well-being.	
**Assessment for each GRADE-CERQual component**	
Methodological limitations	Minor concerns due to poor or partial reporting of the setting in 1 study and limited evidence of sensitivity to ethical concerns in 1 study.
Coherence	No or very minor concerns (the finding describes the key patterns in the data and is well supported by details in the contributing studies).
Relevance	No or very minor concerns regarding relevance (studies include a sufficient range of participants, settings in South Africa and interventions or programmes). While no studies focused on the interventions for pre-term or LBW infants specifically (indirect evidence) we do not anticipate there to be significant differences between pre-term/LBW infants and infants that are not pre-term/LBW that would reduce our confidence in relation to this finding.
Adequacy	Minor concerns (3 studies contributing a fair amount of data)
**Overall GRADE-CERQual assessment and explanation**	
High confidence	

**Contributing studies**	
(37, 39, 48)	
**Finding 14: Training, supervision, and support**<br>LHWs of home visiting programmes have many gaps in the necessary knowledge and skills due to inadequate training, supervision, and support. These deficits make it difficult for LHWs to perform their tasks and may in turn undermine programme credibility and community acceptance. Increased training, supervision and support may positively impact on LHWs' ability to carry out their work due to the increased knowledge, confidence, motivation and sense of accountability it may generate.	
**Assessment for each GRADE-CERQual component**	
Methodological limitations	Minor concerns due to poor or partial reporting of the setting, sampling, data collection and analysis methods in 1 study and limited evidence of reflexivity in 3 of the studies.
Coherence	No or very minor concerns (the finding describes the key patterns in the data and is well supported by details in the contributing studies).
Relevance	No or very minor concerns regarding relevance (studies include a sufficient range of participants, settings in South Africa and interventions or programmes). While no studies focused on the interventions for pre-term or LBW infants specifically (indirect evidence) we do not anticipate there to be significant differences between pre-term/LBW infants and infants that are not pre-term/LBW that would reduce our confidence in relation to this finding.
Adequacy	No or very minor concerns (7 studies contributing a sufficient amount of data)
**Overall GRADE-CERQual assessment and explanation**	
High confidence	
**Contributing studies**	
(39, 40, 42, 43, 48, 50, 53)	
**Finding 15: Practical and logistical challenges**<br>Many LHWS face various practical and logistical challenges when conducting home visits, including inadequate transportation and essential tools and equipment, mobility of clients, and personal safety issues. These challenges make it difficult for LHWs to perform their tasks and may in turn undermine programme credibility and community acceptance.	
**Assessment for each GRADE-CERQual component**	
Methodological limitations	Minor concerns due to poor or partial reporting of the setting, sampling, data collection and analysis methods in 1 study and limited evidence of reflexivity in most of the studies.
Coherence	No or very minor concerns (the finding describes the key patterns in the data and is well supported by details in the contributing studies).
Relevance	No or very minor concerns regarding relevance (studies include a sufficient range of participants, settings in South Africa and interventions or programmes). While no studies focused on the interventions for pre-term or LBW infants specifically (indirect evidence) we do not anticipate there to be significant differences between pre-term/LBW infants and infants that are not pre-term/LBW that would reduce our confidence in relation to this finding.
Adequacy	No or very minor concerns (7 studies contributing a sufficient amount of data)
**Overall GRADE-CERQual assessment and explanation**	
High confidence	
**Contributing studies**	
(38, 39, 42, 43, 49, 50, 53)	
**Finding 16: Human resource-related issues**	

Human resource-related issues, including poor salaries, non-permanent contracts and increased workloads may contribute to the high turnover and attrition rates amongst LHWs in home visiting programmes.	
Assessment for each GRADE-CERQual component	
Methodological limitations	Minor concerns due to poor or partial reporting of the sampling, data collection and analysis methods in 1 study and limited evidence of reflexivity in 2 studies.
Coherence	Minor concerns because some aspects of the review finding were insufficiently described (high turnover and attrition rates; increased workloads) (ambiguous data).
Relevance	No or very minor concerns regarding relevance (studies include a sufficient range of participants, settings in South Africa and interventions or programmes). While no studies focused on the interventions for pre-term or LBW infants specifically (indirect evidence) we do not anticipate there to be significant differences between pre-term/LBW infants and infants that are not pre-term/LBW that would reduce our confidence in relation to this finding.
Adequacy	Moderate concerns (4 studies contributing relatively thin amounts of data)
**Overall GRADE-CERQual assessment and explanation**	
Moderate confidence	Finding downgraded because of minor concerns about methodological limitations, minor concerns about coherence (ambiguous data), and moderate concerns about adequacy.
**Contributing studies**	
(39, 42, 49, 50)	
