# Supplementary material for: Home visits for preterm/low birthweight infants in South Africa: Qualitative evidence synthesis
Source: Afr J Prim Health Care Fam Med. Author manuscript; Available in PMC 2024 Dec 5. (PMC7616882; doi:10.4102/phcfm.v16i1.4701)
Supplement: Supplementary file 1 [file EMS200333-supplement-Appendices.zip › document-1.pdf]

Note: This is Online Appendix 1 of Cooper S, Kallon II, Mabetha D, et al. Home visits for preterm/low birthweight infants in South Africa: Qualitative evidence synthesis. Afr J Prm Health Care Fam Med. 2024;16(1), a4701. https://doi.org/10.4102/phcfm.v16i1.4701

Supplementary material 1. Search strategies

Pubmed (15 September 2023)

Search number	Query	Search Details	Results
8	#1 AND #2 AND #3 AND #4 AND #6 AND #7	("infant, newborn"[MeSH Terms] OR "newborn*"[Title/Abstract] OR "neonate*"[Title/Abstract] OR "baby"[Title/Abstract] OR "babies"[Title/Abstract] OR "child*"[Title/Abstract]) AND ("mother"[Title/Abstract] OR "father"[Title/Abstract] OR "parent*"[Title/Abstract] OR "caregiver"[Title/Abstract] OR "maternal care"[Title/Abstract] OR "famil*"[Title/Abstract] OR "carer"[Title/Abstract] OR "caregivers"[MeSH Terms] OR "mothers"[MeSH Terms] OR "fathers"[MeSH Terms] OR "family"[MeSH Terms] OR "parents"[MeSH Terms] OR "parenting"[MeSH Terms] OR "mother child relations"[MeSH Terms]) AND ("community support"[MeSH Terms] OR "peer influence"[MeSH Terms] OR "peer"[Title/Abstract] OR "Support"[Title/Abstract] OR ("peer"[All Fields] AND ("Support"[All Fields] OR "support s"[All Fields] OR "supported"[All Fields] OR "supporter"[All Fields] OR "supporter s"[All Fields] OR "supporters"[All Fields] OR "supporting"[All Fields] OR "supportive"[All Fields] OR "supportiveness"[All Fields] OR "supports"[All Fields])) OR "help"[Title/Abstract] OR "Assistance"[Title/Abstract] OR "education"[Title/Abstract] OR "mentor mother"[Title/Abstract] OR "home visit*"[Title/Abstract]) AND ("community health services"[MeSH Terms] OR "community health service*"[Title/Abstract] OR "community healthcare"[Title/Abstract] OR "community health care"[Title/Abstract] OR "maternal child health services"[MeSH Terms] OR "maternal child health service*"[Title/Abstract]) AND ("South"[All Fields] AND "africa*"[All Fields]) AND ("qualitative research"[MeSH Terms] OR "interviews as topic"[MeSH Terms] OR "qualitative"[Title/Abstract] OR "group discussion*"[Title/Abstract] OR "focus group*"[Title/Abstract] OR "themes"[Title/Abstract] OR "mixed method*"[Title/Abstract])	179
7	Qualitative research[mh] OR interviews as topic[mh] OR qualitative[tiab] OR group discussion*[tiab] OR focus group*[tiab] OR themes[tiab] OR mixed method*[tiab]	"qualitative research"[MeSH Terms] OR "interviews as topic"[MeSH Terms] OR "qualitative"[Title/Abstract] OR "group discussion*"[Title/Abstract] OR "focus group*"[Title/Abstract] OR "themes"[Title/Abstract] OR "mixed method*"[Title/Abstract]	478,321
6	South Africa*	"South"[All Fields] AND "africa*"[All Fields]	194,524

5	#1 AND #2 AND #3 AND #4	("infant, newborn"[MeSH Terms] OR "newborn*"[Title/Abstract] OR "neonate*"[Title/Abstract] OR "baby"[Title/Abstract] OR "babies"[Title/Abstract] OR "child*"[Title/Abstract]) AND ("mother"[Title/Abstract] OR "father"[Title/Abstract] OR "parent*"[Title/Abstract] OR "caregiver"[Title/Abstract] OR "maternal care"[Title/Abstract] OR "famil*"[Title/Abstract] OR "carer"[Title/Abstract] OR "caregivers"[MeSH Terms] OR "mothers"[MeSH Terms] OR "fathers"[MeSH Terms] OR "family"[MeSH Terms] OR "parents"[MeSH Terms] OR "parenting"[MeSH Terms] OR "mother child relations"[MeSH Terms]) AND ("community support"[MeSH Terms] OR "peer influence"[MeSH Terms] OR "peer"[Title/Abstract] OR "Support"[Title/Abstract] OR ("peer"[All Fields] AND ("Support"[All Fields] OR "support s"[All Fields] OR "supported"[All Fields] OR "supporter"[All Fields] OR "supporter s"[All Fields] OR "supporters"[All Fields] OR "supporting"[All Fields] OR "supportive"[All Fields] OR "supportiveness"[All Fields] OR "supports"[All Fields])) OR "help"[Title/Abstract] OR "Assistance"[Title/Abstract] OR "education"[Title/Abstract] OR "mentor mother"[Title/Abstract] OR "home visit*"[Title/Abstract]) AND ("community health services"[MeSH Terms] OR "community health service*"[Title/Abstract] OR "community healthcare"[Title/Abstract] OR "community health care"[Title/Abstract] OR "maternal child health services"[MeSH Terms] OR "maternal child health service*"[Title/Abstract])	16,456
4	Community Health Services [Mesh] OR Community Health Service*[tiab] OR community healthcare [tiab] OR community health care [tiab] OR Maternal-Child Health Services [Mesh] OR Maternal Child Health Service*[tiab]	"community health services"[MeSH Terms] OR "community health service*"[Title/Abstract] OR "community healthcare"[Title/Abstract] OR "community health care"[Title/Abstract] OR "maternal child health services"[MeSH Terms] OR "maternal child health service*"[Title/Abstract]	335,745
3	Community support [Mesh] OR Peer influence [Mesh] OR peer [tiab] OR Support [tiab] OR peer support OR help [tiab] OR Assistance [tiab] OR education [tiab] OR mentor mother [tiab] OR home visit* [tiab]	"community support"[MeSH Terms] OR "peer influence"[MeSH Terms] OR "peer"[Title/Abstract] OR "Support"[Title/Abstract] OR ("peer"[All Fields] AND ("Support"[All Fields] OR "support s"[All Fields] OR "supported"[All Fields] OR "supporter"[All Fields] OR "supporter s"[All Fields] OR "supporters"[All Fields] OR "supporting"[All Fields] OR "supportive"[All Fields] OR "supportiveness"[All Fields] OR "supports"[All Fields])) OR "help"[Title/Abstract] OR "Assistance"[Title/Abstract] OR "education"[Title/Abstract] OR "mentor mother"[Title/Abstract] OR "home visit*"[Title/Abstract]	2,556,887
2	mother [tiab] OR father [tiab] OR parent* [tiab] OR caregiver [tiab] OR "maternal care" [tiab] OR famil* [tiab] OR carer [tiab] OR Caregivers [Mesh] OR Mothers [Mesh] OR Fathers [Mesh] OR Family [Mesh] OR Parents [Mesh] OR Parenting [Mesh] OR Mother-Child Relations [Mesh]	"mother"[Title/Abstract] OR "father"[Title/Abstract] OR "parent*"[Title/Abstract] OR "caregiver"[Title/Abstract] OR "maternal care"[Title/Abstract] OR "famil*"[Title/Abstract] OR "carer"[Title/Abstract] OR "caregivers"[MeSH Terms] OR "mothers"[MeSH Terms] OR "fathers"[MeSH Terms] OR "family"[MeSH Terms] OR "parents"[MeSH Terms] OR "parenting"[MeSH Terms] OR "mother child relations"[MeSH Terms]	1,998,286
1	Infant, newborn [Mesh] OR Newborn*[tiab] OR neonate*[tiab] OR baby [tiab] OR babies [tiab] OR Child*[tiab]	"infant, newborn"[MeSH Terms] OR "newborn*"[Title/Abstract] OR "neonate*"[Title/Abstract] OR "baby"[Title/Abstract] OR "babies"[Title/Abstract] OR "child*"[Title/Abstract]	2,308,287

Search strategy for Embase via Ovid (15 September 2023)

No.	Query	Results
#2	(('community care'/exp OR 'community care' OR 'home visit'/exp OR 'home visit' OR 'community health service*':ab,ti OR (('community*based' NEAR/3 (service* OR care)):ab,ti)) AND ('south africa'/exp OR 'south africa' OR 'south african'/exp OR 'south african' OR 'south africa':ab,ti OR 'south african':ab,ti) AND ('qualitative research'/exp OR 'qualitative research' OR 'interview'/exp OR interview OR 'focus group'/exp OR 'focus group' OR qualitative:ab,ti OR interview*:ab,ti OR 'focus group*':ab,ti OR theme*:ab,ti) AND ('maternal care'/exp OR 'maternal care' OR 'newborn care'/exp OR 'newborn care' OR 'postnatal care'/exp OR 'postnatal care' OR 'perinatal care'/exp OR 'perinatal care' OR 'maternal child health care'/exp OR 'maternal child health care' OR 'child health care'/exp OR 'child health care' OR 'child health'/exp OR 'child health' OR 'maternal care':ti,ab,kw OR 'newborn care':ti,ab,kw OR 'postnatal care':ti,ab,kw OR 'perinatal care':ti,ab,kw OR 'maternal child health care':ti,ab,kw OR 'child health care':ti,ab,kw OR 'child health':ti,ab,kw OR 'maternal child health service*':ti,ab,kw)) AND ('health auxiliary'/exp OR 'health auxiliary')	24
#1	('community care'/exp OR 'community care' OR 'home visit'/exp OR 'home visit' OR 'community health service*':ab,ti OR (('community*based' NEAR/3 (service* OR care)):ab,ti)) AND ('south africa'/exp OR 'south africa' OR 'south african'/exp OR 'south african' OR 'south africa':ab,ti OR 'south african':ab,ti) AND ('qualitative research'/exp OR 'qualitative research' OR 'interview'/exp OR interview OR 'focus group'/exp OR 'focus group' OR qualitative:ab,ti OR interview*:ab,ti OR 'focus group*':ab,ti OR theme*:ab,ti) AND ('maternal care'/exp OR 'maternal care' OR 'newborn care'/exp OR 'newborn care' OR 'postnatal care'/exp OR 'postnatal care' OR 'perinatal care'/exp OR 'perinatal care' OR 'maternal child health care'/exp OR 'maternal child health care' OR 'child health care'/exp OR 'child health care' OR 'child health'/exp OR 'child health' OR 'maternal care':ti,ab,kw OR 'newborn care':ti,ab,kw OR 'postnatal care':ti,ab,kw OR 'perinatal care':ti,ab,kw OR 'maternal child health care':ti,ab,kw OR 'child health care':ti,ab,kw OR 'child health':ti,ab,kw OR 'maternal child health service*':ti,ab,kw)	97
